# Paternal undernutrition and overnutrition modify semen composition and preimplantation embryo developmental kinetics in mice

**DOI:** 10.1186/s12915-024-01992-0

**Published:** 2024-09-16

**Authors:** Hannah L. Morgan, Nader Eid, Nadine Holmes, Sonal Henson, Victoria Wright, Clare Coveney, Catherine Winder, Donna M. O’Neil, Warwick B. Dunn, David J. Boocock, Adam J. Watkins

**Affiliations:** 1 Lifespan and Population Health, School of Medicine, University of Nottingham, Nottingham, NG7 2UH UK; 2 Deep Seq, School of Life Sciences, Queen’s Medical Centre, University of Nottingham, Nottingham, NG7 2UH UK; 3 The John Van Geest Cancer Research Centre, Nottingham Trent University, Nottingham, NG11 8NS UK; 4 Phenome Centre, School of Biosciences, University of Birmingham, Birmingham, B15 2TT UK

**Keywords:** Paternal diet, Sperm RNA, Semen profile, Embryo development, Uterine response

## Abstract

**Background:**

The importance of parental diet in relation to eventual offspring health is increasing in prominence due to the increased frequency of parents of reproductive age consuming poor diets. Whilst maternal health and offspring outcome have been studied in some detail, the paternal impacts are not as well understood. A father’s poor nutritional status has been shown to have negative consequences on foetal growth and development and ultimately impact the long-term adult health of the offspring. In this study, we examined sperm- and seminal vesicle fluid-mediated mechanisms of preimplantation embryo development alterations in response to sub-optimal paternal diets.

**Results:**

Male mice were fed a diet to model either under (low-protein diet (LPD)) or over (high-fat/sugar ‘Western’ diet (WD)) nutrition, LPD or WD supplemented with methyl donors or a control diet (CD) before mating with age-matched females. Male metabolic health was influenced by WD and MD-WD, with significant changes in multiple serum lipid classes and hepatic 1-carbon metabolites. Sperm RNA sequencing revealed significant changes to mRNA profiles in all groups when compared to CD (LPD: 32, MD-LPD: 17, WD: 53, MD-WD: 35 transcripts). Separate analysis of the seminal vesicle fluid proteome revealed a significant number of differentially expressed proteins in all groups (LPD: 13, MD-LPD: 27, WD: 24, MD-WD: 19) when compared to control. Following mating, in vitro time-lapse imaging of preimplantation embryos revealed a significant increase in the timing of development in all experimental groups when compared to CD embryos. Finally, qPCR analysis of uterine tissue at the time of implantation identified perturbed expression of *Cd14* and *Ptgs1* following mating with WD-fed males.

**Conclusions:**

Our current study shows that paternal nutritional status has the potential to influence male metabolic and reproductive health, impacting on embryonic development and the maternal reproductive tract. This study highlights potential direct (sperm-mediated) and indirect (seminal vesicle fluid-mediated) pathways in which a father’s poor diet could shape the long-term health of his offspring.

**Supplementary Information:**

The online version contains supplementary material available at 10.1186/s12915-024-01992-0.

## Background

Globally, an increasing number of adults are entering reproductive age consuming diets of poor quality, being either excessive in their abundance of fats, sugars and carbohydrates or deficient in protein or micronutrients such as folate. Whilst such lifestyle factors may not directly impede their fertility or chances of becoming parents, studies have shown parental nutrition at the time of conception is critical for shaping patterns of post-fertilisation development, foetal growth and even long-term offspring health [1]. Both maternal overnutrition and undernutrition during gestation have been associated with increased risk for adult non-communicable diseases such as obesity, heart disease and type 2 diabetes in her offspring [2–4]. Observations from distinct epidemiological data sets and animal model studies have indicated that different periods throughout gestation, and even prior to conception, display differential sensitivity to poor parental diet [5]. Women who were in early gestation during the Dutch Hunger Winter gave birth to children of normal weight who went on to become obese and develop coronary artery disease, dyslipidaemia and glucose intolerance in adulthood [6–8]. In mice, maternal diets low in protein [9] or high in fat [10] during the periconception period impair embryo development and metabolism, influencing long-term offspring cardiometabolic ill health and behaviour. Separately, maternal diets with imbalances in key micronutrients, such as low in B vitamins and methionine [11] or high in folate [12] around the time of conception in mice, also impact on embryonic development, foetal growth and epigenetic regulation of gene expression. Similar to the observation in animals, maternal periconception increases in dietary folate, betaine and folic acid intake in women lead to changes in infant DNA methylation of genes involved in metabolism, growth and appetite regulation [13].

Interestingly, preconception increases in methyl donor micronutrient intake in either parent have been found to lead to changes in gene DNA methylation status in infants [13, 14]. These observations indicate that paternal, as well as maternal, diet could influence the post-conception development and well-being of their offspring. It is becoming increasingly evident that the nutritional state of the father at the time of conception also influences embryonic, foetal and postnatal development. Diet-induced obesity in male mice modulates both the transcriptome as well as the epigenetic profile of the mature sperm [15–18]. Separately, male mice fed with diets low in protein [19, 20] or folate [21] also display perturbed sperm epigenetic status. Poor paternal diet also impacts on post fertilisation development influencing preimplantation embryo metabolism [20], foetal growth [19, 21–23] and long-term adult ill-health [24, 25]. Alongside the sperm, diet-induced obesity alters the levels of cytokines, hormones and metabolites within the seminal plasma [15–18]. As seminal plasma components elicit a range of vascular, inflammatory and immune cell responses within the female reproductive tract [26], dietary-induced changes in seminal plasma composition could provide additional mechanisms of offspring programming [20].

Previously, we have shown that a paternal low-protein diet (LPD), with or without methyl donor supplementation, influence placental and foetal development in mice [23, 27]. Our studies also highlight roles for both the sperm and seminal plasma in directing post-fertilisation development [20]. In the current study, we expand on our earlier investigations to define the significance of both paternal under (LPD) and over (high-fat/sugar (Western) diet) nutrition on semen composition and post-fertilisation development. Furthermore, we explore whether supplementation with a mix of methyl donors negates any detrimental influences of these poor-quality diets. We hypothesise that undernutrition and overnutrition will negatively impact paternal reproductive health and influence changes in post-fertilisation development, with the supplementation of methyl donors acting to ameliorate these observed impairments.

## Results

### Male adiposity, liver one-carbon metabolites and lipid profiles are altered by a Western diet and methyl donor supplementation

To define the impact of undernutrition and overnutrition, as well as the impact of methyl donor supplementation, we fed male C57BL/6 J mice either a control (CD; 18% casein, 21% sugar, 0% milk fat, 0% cholesterol [0.74 kcal/g protein, 1.80 kcal/g carbohydrates, 0.56 kcal/g fat]), isocaloric low-protein diet (LPD; 9% casein, 24% sugar, 0% milk fat, 0% cholesterol [0.37 kcal/g protein, 2.17 kcal/g carbohydrates, 0.56 kcal/g fat]), ‘Western’ diet (WD; 19% casein, 34% sugar, 20% milk fat, 0.15% cholesterol [0.70 kcal/g protein, 2.00 kcal/g carbohydrates, 1.93 kcal/g fat]) or LPD or WD supplemented with methyl donors (an addition of 5 g/kg diet choline chloride, 15 g/kg diet betaine, 7.5 g/kg diet methionine, 15 mg/kg diet folic acid, 1.5 mg/kg diet vitamin B_12_; termed MD-LPD and MD-WD respectively).

**Fig. 1:**
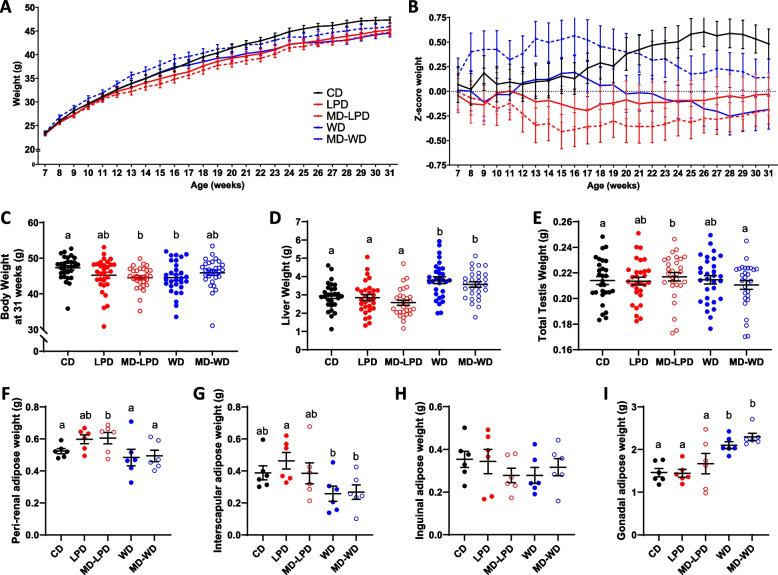
The impact of paternal diet on male physiology. Male weekly weight progression (**A**) and *Z*-score of weight change (**B**) over 24 weeks of diet exposure. Male post-mortem body (**C**), liver (**D**) and testis (**E**) weights at end of study period; CD *n* = 28, LPD *n* = 30, MD-LPD *n* = 29, WD *n* = 30, MD-WD *n* = 28. The weight of different fat pads was examined in a sub-set of males (*n* = 6 for all groups): the peri-renal (**F**), inter-scapular (**G**), inguinal (**H**) and gonadal (**I**). Data presented as mean ± SEM. Statistical significance determined using one-way ANOVA with Tukey’s post-test. Different letters denote significance; *p* < 0.05

Over a 24-week feeding period, no significant differences in overall stud male growth profiles were observed between any of the dietary groups (Fig. 1A, B). However, at the time of cull, MD-LPD and WD males displayed a significantly reduced mean body weight when compared to CD males (Fig. 1C; *p* < 0.05). WD and MD-WD males displayed an increase in relative liver weight, compared to all other dietary groups (Fig. 1D; *p* < 0.00001), whilst MD-LPD males displayed an increase in relative total testes weight compared to CD (Fig. 1E; *p* < 0.05). Analysis of adiposity in a subset of males (*n* = 6) revealed MD-LPD-fed males had a significant increase in relative peri-renal adipose tissue weight (Fig. 1F; *p* < 0.01) compared to CD males, and WD- and MD-WD-fed males had a reduced relative interscapular adipose weight (Fig. 1G; *p* < 0.05) compared to LPD males. There were no differences observed in relative weight of the inguinal fat mass (Figs. 1H); however, WD and MD-WD males displayed a relative increase in gonadal fat (Fig. 1I; *p* < 0.01 and *p* < 0.001 respectively), compared to CD males.

**Fig. 2:**
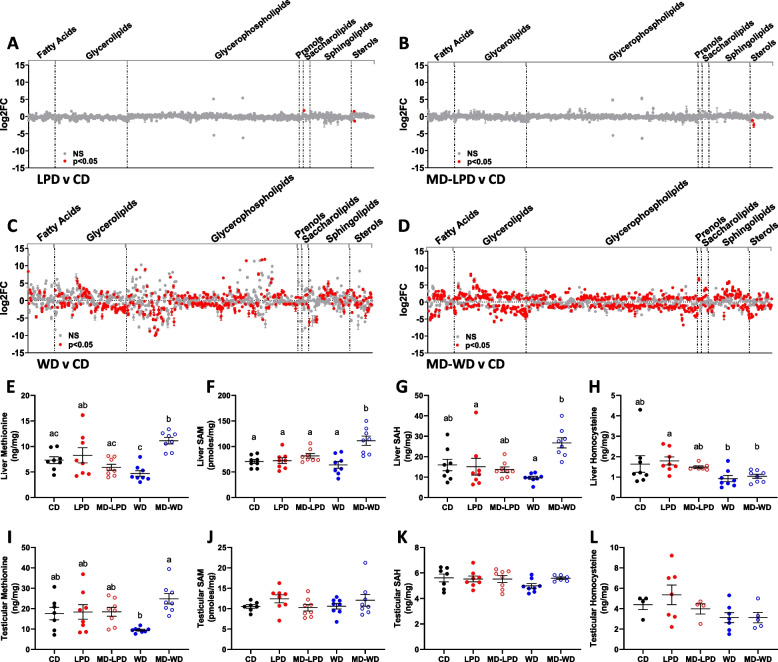
The impact of paternal diet on male metabolic status. Differential serum lipid abundance in (**A**) LPD males, (**B**) MD-LPD males, (**C**) WD males and (**D**) MD-WD males when compared to CD males. Hepatic levels of the central one-carbon metabolites (**E**) methionine, (**F**) S-adenosylmethionine (SAM), (**G**) S-adenosylhomocysteine (SAH) and (**H**) homocysteine. Testicular levels of the central one-carbon metabolites (**I**) methionine, (**J**) S-adenosylmethionine (SAM), (**K**) S-adenosylhomocysteine (SAH) and (**L**) homocysteine. (**E**–**L**) Data presented as individual male values with mean ± SEM; statistical significance determined using one-way ANOVA with Tukey’s post-test, different letters denote significance *p* < 0.05. *n* = 8 males per diet group

Lipidomic analysis of the stud male serum after 24 weeks of diet exposure revealed relatively few changes in the number of differentially abundant lipids between CD-, LPD- and MD-LPD-fed males (Fig. 2A, B). Specifically, N-oleoyl phenylalanine and 26-hydroxycholesterol 3-sulphate were increased in their abundance in LPD males (1.76 and 1.51 log_2_ fold respectively; *p* < 0.001), whilst 24-methylene-cholesterol sulphate was decreased in abundance (− 1.36 log_2_ fold, *p* < 0.001). In MD-LPD males, only 24-methylene-cholesterol sulphate and calcidiol were significantly decreased in abundance (− 1.12 and − 2.51 log_2_ fold respectively, *p* < 0.001). In contrast, males fed either WD or MD-WD displayed 367 and 660 differentially abundant lipids respectively when compared to CD males (Fig. 2C, D; *p* < 0.05). Both WD and MD-WD males displayed increased and decreased abundance of a range of lipid classes including fatty acids, glycerolipids, glycerophospholipids, prenols, saccharolipids, sphingolipids and sterols. Interestingly, whilst no lipids were differentially abundant between direct comparisons of WD and MD-WD males, WD males displayed differential abundance of 24 lipids compared to CD males that were not differential in the MD-WD males compared to CD, whilst MD-WD males displayed 273 differential lipids that were not altered in WD males when compared to CD. However, we did observe greater variability between biological samples in WD-fed male serum (outlined in Additional file 1: Fig. S1) which may impact the certainty of these interpretations. The full details of all lipid changes, including probabilistic quotient normalisation values for each metabolite and each biological replicate, can be found in Additional file 2: Table S1.

We additionally conducted targeted metabolomics analysis of central one-carbon metabolites in liver and testis tissue of our stud males after 24 weeks of diet exposure. One-carbon metabolism is a fundamental metabolic process which functions to deliver methyl-groups from dietary components for use in multiple physiological processes and cellular reactions. Determination of the key metabolites from these pathways gives insight into tissue nutritional status. We found no significant difference between liver metabolites of LPD males compared to MD-LPD males, with both showing no significant change compared to CD males (Fig. 2E–H). WD males displayed decreased levels of liver methionine (Fig. 2E; *p* < 0.0001), S-adenosylmethionine (SAM) (Fig. 2F; *p* < 0.0001) and S-adenosylhomocysteine (SAH) (Fig. 2G; *p* = 0.0006) compared to MD-WD males; however, liver levels of homocysteine were unchanged between WD and MD-WD (Fig. 2H). MD-WD also displayed elevated methionine (Fig. 2E; *p* = 0.0289) and S-adenosylhomocysteine (SAH) (Fig. 2G; *p* = 0.0004) compared to CD males. In the testis, the WD males displayed a significant decrease in the amounts of methionine relative to MD-WD males (Fig. 2I; *p* = 0.0013); however, this was not significantly different to the amount observed in CD males. No other difference in testicular 1-carbon metabolites were observed across groups (Fig. 2J - L).

**Fig. 3:**
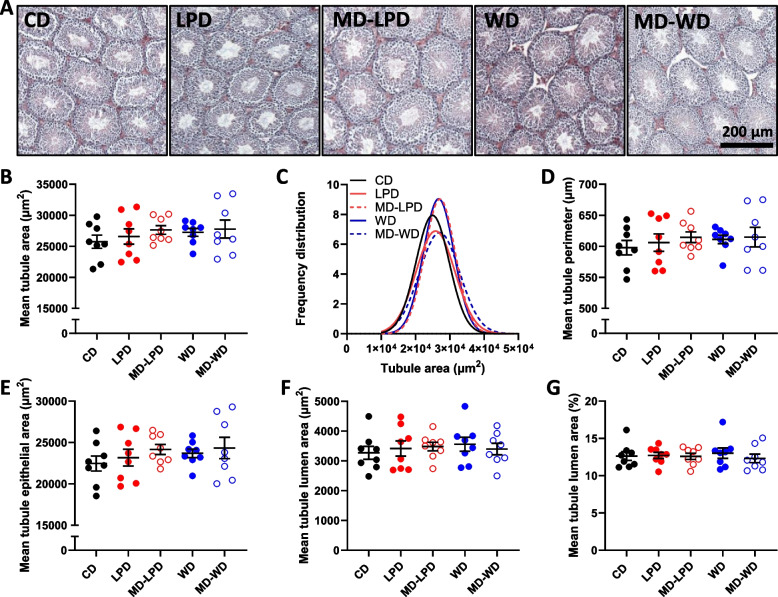
The impact of paternal diet on testicular morphology. **A** Representative histological images of testes from CD, LPD, MD-LPD, WD and MD-WD males. **B** Mean seminiferous tubule cross section area and (**C**) frequency distribution of tubule area. Mean seminiferous tubule (**D**) perimeter, (**E**) area of the epithelium, (**F**) area of the tubule lumen and (**G**) % area of the lumen. *n* = 8 males per diet group

Histological analysis of the testis (Fig. 3A) revealed no difference in mean seminiferous tubule cross sections area (Fig. 3B) or frequency of tubule area (Fig. 3C), tubule perimeter (Fig. 3D), area of the seminiferous epithelium (Fig. 3E) or the area of the tubule lumen (Fig. 3F, G) across groups.

### Sub-optimal paternal diets altered the sperm RNA profiles

**Fig. 4:**
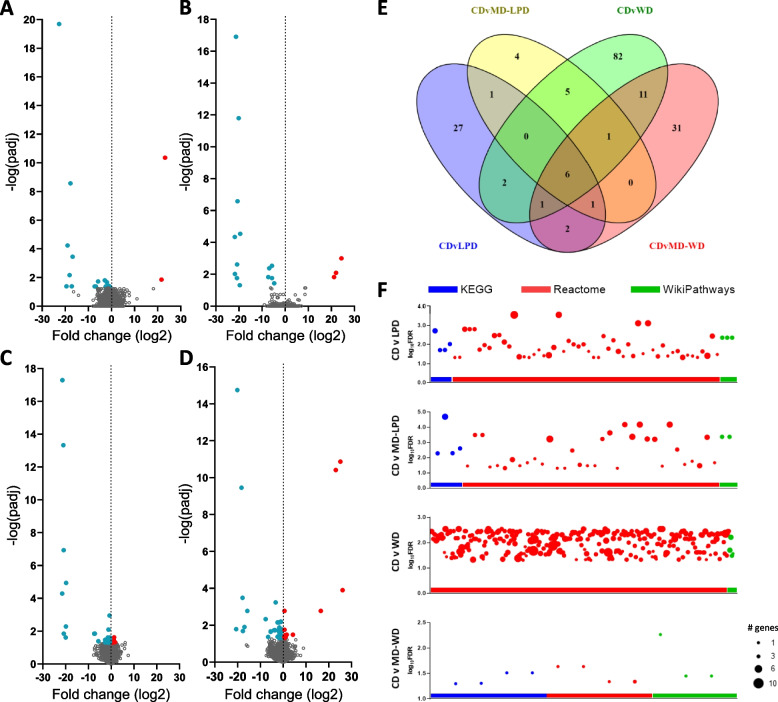
Sub-optimal paternal diet modifies the sperm transcriptome. Volcano plots outlining the number of significantly up (red) and down (blue) differentially expressed protein coding genes (DEGs) detected in sperm from (**A**) LPD, (**B**) MD-LPD, (**C**) WD and (**D**) MD-WD males compared to CD sperm. **E** Venn diagram outlining the total number of DEGs in each comparison group and the overlap between all comparisons. Constructed using Venny 2.1 [28]. **F** The number of pathways predicted to be influenced by the sperm DEGs in each diet group compared to CD. Each dot represents one pathway, colour-coded for pathway database and size coded for the number of DEGs within that pathway. *n* = 6 males per diet group

Analysis of sperm mRNA content revealed significant changes in expression of multiple sperm protein coding genes in all groups when compared to CD (LPD: 32, MD-LPD: 17, WD: 53, MD-WD: 35, Fig. 4A–D). Analysis of the overlap in differentially expressed genes (DEGs) of all biotypes between diets was examined using Venny 2.1 [28] and revealed 27 unique mRNA in LPD sperm, 4 in MD-LPD sperm, 82 in WD sperm and 31 in MD-WD sperm (Fig. 4E; full details of all significant DEGs in Additional file 3: Table S2, full RNASeq details in GEO:GSE241404). Six DEGs were found to be differentially expressed in all diet groups, *Gm43064*, *Apoa1*, *Pcdhga10*, *Apoa4*, *Gc* and *Fgg*, all demonstrating an increase in relative expression. Pathway analysis was conducted on the identified protein coding DEGs using the ShinyGO web-based software to highlight the significantly impacted pathways (FDR < 0.05, determined using KEGG, Reactome and WikiPathways databases) and the number of genes involved (Fig. 4F). Sperm DEGs from LPD-, MD-LPD- and MD-WD-fed fathers were predicted to influence 61, 40 and 10 pathways, respectively, whereas the sperm DEGs from WD-fed fathers predicted changes to 296 pathways. Pathways involving lipoprotein remodelling and assembly (R-MMU-174824.1) were identified in all diet groups. Pathways involved in DNA replication and synthesis, as well as pathways involved in cell cycle control (mainly due to changes in *Ubc* and *Ubb*), were identified only in DEGs from WD sperm. All predicted pathways are detailed in Additional file 4: Table S3.

### Sub-optimal paternal diets accelerated embryo development in vitro

**Fig. 5:**
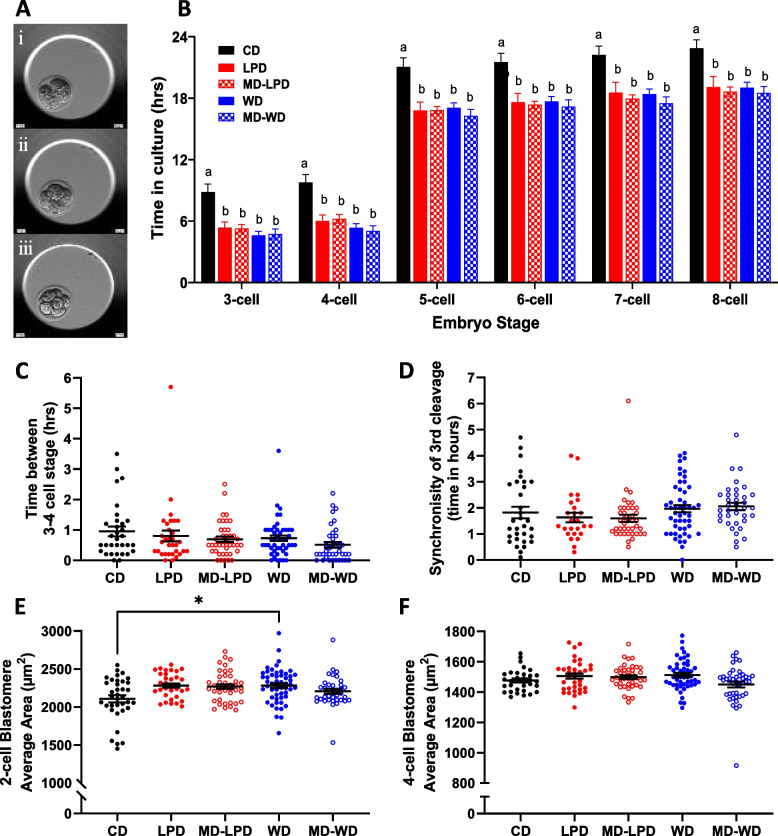
Sub-optimal paternal diet enhances pre-compaction preimplantation embryo kinetics. **A** Representative time lapse images of a (i) 2-cell, (ii) 4-cell and (iii) 8-cell embryo. **B** Preimplantation embryo development timings of pre-compaction cleavage stages normalised to first cell cleavage. **C** Cleavage time between 3 and 4 cell and (**D**) synchronicity of the 3rd cleavage (4-cell–8-cell) for each individual embryo. Blastomere area was measured and averaged for each embryo at the (**E**) 2-cell stage and (**F**) 4-cell stage. Data presented as mean ± SEM in **B** and as individual embryos in **C**–**F**. CD *n* = 36 embryos from 5 litters, LPD *n* = 45 embryos from 6 litters, MD-LPD *n* = 42 embryos from 6 litters, WD *n* = 51 embryos from 7 litters, MD-WD *n* = 42 embryos from 6 litters (each litter generated by a separate male). Statistical significance determined using generalised linear mixed model analysis factored for stud male; different letters denote statistical significance or *p<0.05

To determine the sperm’s impact on directing embryo development, we collected 2-cell embryos on embryonic day 1.5 (E1.5) and cultured them in vitro to exclude any maternal or seminal plasma influences. We observed no differences in the number of embryos retrieved at E1.5 (Additional file 5: Table S4). Embryo development was continually recorded during the 60 h culture period using an EmbryoScope time-lapse incubator (Fig. 5A; representative images of pre-compaction stages). In vitro time-lapse assessment of embryo development revealed embryos from all experimental dietary groups progressed through the pre-compaction cleavage stages faster than CD embryos (Fig. 5B). Analysis of the time taken to progress from the 3- to the 4-cell stage did not differ (Fig. 5C), and there was no difference in the synchronicity of the 3rd cleavage stage between groups (Fig. 5D). Analysis of blastomere size at the 2-cell stage revealed embryos from WD-fed males had a significantly larger mean area when compared to CD embryos (Fig. 5E, *p* = 0.0286). However, this difference in size was not maintained at the 4-cell stage (Fig. 5F).

**Fig. 6:**
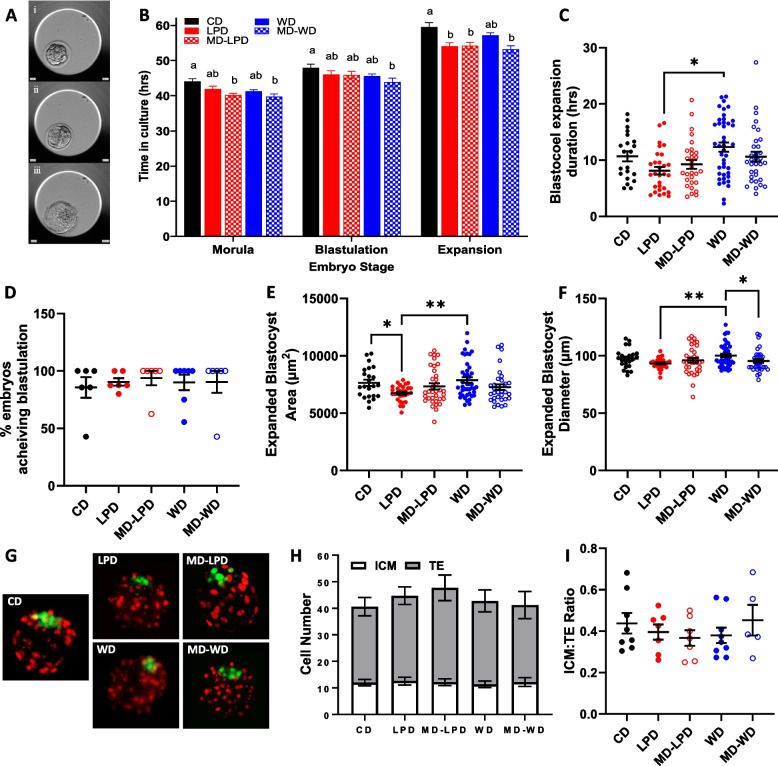
Sub-optimal paternal diet enhances post-compaction preimplantation embryo kinetics. **A** Representative time lapse images of a (i) morula, (ii) early blastocyst and (iii) an expanded blastocyst. **B** Preimplantation embryo development timings of post-compaction cleavage stages. **C** Blastocyst expansion time, from first appearance of blastocoel cavity to full expansion, and (**D**) the percentage of the litter that achieved blastulation. **E** Area and (**F**) diameter of the fully expanded blastocysts. **G** Representative composite images of whole blastocysts stained with Cdx2 (trophectoderm = red) and Oct4 (inner cell mass = green) for the determination of lineage cell allocation. **H** The absolute number of inner cell mass (ICM), trophectoderm (TE) cells and total cell number of stained blastocysts. **I** Ratio of the ICM to TE cell number. Data presented as mean ± SEM in **B**, **D**, **H** and as individual embryos in **C**, **E**, **F**, **I**. **B**, **C**, **D**, **E**, **F** CD *n* = 36 embryos from 5 litters, LPD *n* = 45 embryos from 6 litters, MD-LPD *n* = 42 embryos from 6 litters, WD *n* = 51 embryos from 7 litters, MD-WD *n* = 42 embryos from 6 litters. **H**, **I** CD *n* = 8 embryos from 5 litters, LPD *n* = 7 embryos from 6 litters, MD-LPD *n* = 7 embryos from 6 litters, WD *n* = 9 embryos from 7 litters, MD-WD *n* = 5 embryos from 6 litters. Each litter generated by a separate male. Statistical significance determined using generalised linear mixed model analysis factored for stud male; different letters denote statistical significance or *p < 0.05, **p < 0.01

Post-compaction (representative staging shown in Fig. 6A), whilst embryos from the MD-LPD- and MD-WD-fed males developed to the morula stage significantly faster than CD embryos (Fig. 6B; *p* < 0.05), embryos from LPD- and WD-fed males were no longer more advanced. However, the time taken for LPD-, MD-LPD- and MD-WD-derived embryos to reach full expansion was again significantly reduced when compared to CD derived embryos (Fig. 6B; *p* < 0.05). Furthermore, there was an increased expansion time observed for WD embryos compared to LPD (Fig. 6C; *p* = 0.036). We observed no difference in the percentage of embryos reaching either the blastocyst (Fig. 6D), or expanded blastocyst stage (Additional file 5: Table S4), between diet groups. We also saw no difference in the proportion of embryos undergoing developmental arrest, either pre- or post-blastulation (Additional file 5: Table S4). After 60 h in culture, there was an observed decrease in the expanded blastocyst area in embryos from LPD-fed males, compared to CD and WD (Fig. 6E; *p* = 0.025 and 0.001, respectively). This change in LPD was reflected by a decrease in blastocyst diameter compared to WD (Fig. 6F; *p* = 0.003). Whilst MD-WD and WD blastocyst area was not significantly different, WD demonstrated an increased blastocyst diameter when compared to MD-WD blastocysts (Fig. 6F; *p* = 0.031). However, assessment of blastocysts cell numbers, determined by Cdx2 and Oct4 staining to identify the trophectoderm (TE) and inner cell mass (ICM) respectively (Fig. 6G), showed no differences in total, ICM or TE cell numbers (Fig. 6H) or in the ratio of the two cell lineage types (Fig. 6I).

### Paternal diet alters the seminal vesicle fluid proteome

**Fig. 7:**
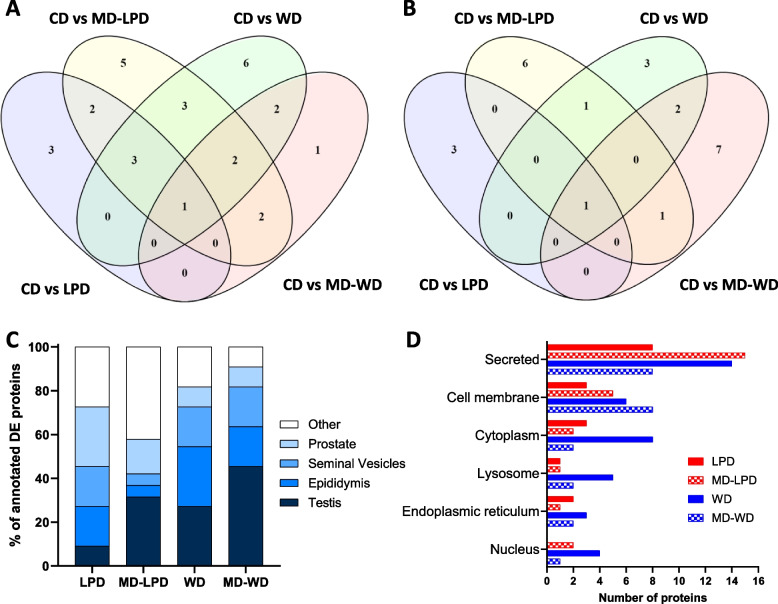
The pattern and tissue and sub-cellular locations of differentially expressed seminal vesicle fluid proteins. **A** The number of seminal vesicle fluid proteins found to increase in response to sub-optimal diets compared to CD and overlap between different dietary groups. **B** The number of seminal vesicle fluid proteins found to decrease in response to sub-optimal diets compared to CD and overlap between different dietary groups. Venn diagrams constructed using Venny 2.1 [28]. **C** UniProt-annotated predicted tissue specific locations of differentially expressed seminal vesicle fluid proteins. **D** The subcellular locations of the differentially expressed seminal vesicle fluid proteins compared to control. *n* = 6/diet group

We have previously shown that offspring development and well-being can be programmed through both sperm- and seminal fluid-mediated mechanisms [20, 29, 30]. Therefore, under this study, we also examined the protein composition of the seminal vesicle fluid in response to paternal sub-optimal diet. Across all males, a total of 235 proteins were detected in at least 3 seminal vesicle fluid sample replicates (see Additional file 6: Table S5 for full protein count details, including both unique and shared protein groups based on peptides used for quantitation, and PRIDE submission: PXD044980 for full datasets). Using StatsPro software with CV threshold of 0.5 and no fold change threshold, we identified a number of seminal vesicle fluid proteins that were significantly (*padj* < 0.05) differentially expressed in their abundance in response to the different dietary regimens (Table 1; full limma outputs available in Additional file 7: Table S6). A significant upregulation of multiple inositol polyphosphate phosphatase 1 and downregulation of NPC intracellular cholesterol transporter 2 was consistently observed in all sub-optimal diet groups compared to CD (Table 1, Fig. 7A, B). Few proteins displayed diet-specific differential profiles with five proteins different from CD in LPD and MD-LPD groups (biotinidase, probable G-protein coupled receptor 132, protein FAM3B, cystatin-C and alpha-amylase 1; all upregulated (Fig. 7A), when compared to CD) and six proteins different in both WD and MD-WD groups compared to CD. These were four upregulated proteins (transcobalamin-2, beta-2-microglobulin, alpha-1-acid glycoprotein 3 and seminal vesicle secretory protein 4) (Table 1 and Fig. 7A) and two downregulated proteins (major prion protein, and UDP-GlcNAc:betaGal beta-1,3-N-acetylglucosaminyltransferase 7) (Table 1 and Fig. 7B). Interestingly, three proteins were altered based solely on methyl donor supplementation (common changes in CD vs MD-LPD and CD vs MD-WD). These were an upregulation of E3 ubiquitin-protein ligase BRE1B and seminal vesicle secretory protein 6 and a downregulation of angiotensin-converting enzyme (Table 1 and Fig. 7A, B).

**Table 1:** Log2Fold change of significant seminal vesicle fluid protein abundance changes compared to CD

UniProt ID	Gene symbol	Gene name	LPD	MD-LPD	WD	MD-WD
Q60590	Orm1	Alpha-1-acid glycoprotein 1	-	0.55	0.52	0.32
Q63805	Orm3	Alpha-1-acid glycoprotein 3	-	-	0.75	-
P00688;P00687	Amy1	Alpha-amylase 1	0.69	0.72	1.02	-
P27046	Man2a1	Alpha-mannosidase 2	-	− 0.48	-	-
P09470	Ace	Angiotensin-converting enzyme	-	− 0.26	-	− 0.32
P01887	B2m	Beta-2-microglobulin	-	-	0.38	0.37
P23780	Glb1	Beta-galactosidase	-	− 0.44	-	-
Q8K2I4	Manba	Beta-mannosidase	-	-	-	0.50
Q8CIF4	Btd	Biotinidase	0.39	0.40	-	-
P09803	Cdh1	Cadherin-1	-	0.38	-	-
Q80V42	Cpm	Carboxypeptidase M	-	− 0.33	-	-
P10605	Ctsb	Cathepsin B	-	-	0.30	-
P18242	Ctsd	Cathepsin D	-	− 0.68	-	-
Q9R013	Ctsf	Cathepsin F	-	-	-	− 0.64
Q9WUU7	Ctsz	Cathepsin Z	-	-	-	− 0.49
P21460	Cst3	Cystatin-C	0.31	0.37	0.34	-
P56542	Dnase2	Deoxyribonuclease-2-alpha	-	-	0.61	-
Q8R242	Ctbs	Di-N-acetylchitobiase	-	-	0.34	-
Q3U319	Rnf40	E3 ubiquitin-protein ligase BRE1B	-	0.51	-	0.26
P05064	Aldoa	Fructose-bisphosphate aldolase A	-	-	− 0.53	-
Q07235	Serpine2	Glia-derived nexin	-	0.18	-	-
Q60928	Ggt1	Glutathione hydrolase 1 proenzyme	-	0.75	-	-
P15626	Gstm2	Glutathione S-transferase Mu 2	− 0.68	-	-	-
Q9Z0M9	Il18bp	Interleukin-18-binding protein	-	− 0.59	− 0.58	-
P04104	Krt1	Keratin, type II cytoskeletal 1	− 0.83	-	-	-
Q6NXH9	Krt73	Keratin, type II cytoskeletal 73	− 0.91	-	-	-
Q7M6Z4	Kif27	Kinesin-like protein KIF27	-	-	-	− 0.49
O09159	Man2b1	Lysosomal alpha-mannosidase	-	-	− 0.41	-
P04925	Prnp	Major prion protein	-	-	− 0.64	− 0.77
P12032	Timp1	Metalloproteinase inhibitor 1	0.49	-	-	-
**Q9Z2L6**	**Minpp1**	**Multiple inositol polyphosphate phosphatase 1**	**0.76**	**0.65**	**0.98**	**0.52**
Q8BFR4	Gns	N-acetylglucosamine-6-sulfatase	-	-	0.53	-
Q6ZWR6	Syne1	Nesprin-1	-	0.74	0.74	-
**Q9Z0J0**	**Npc2**	**NPC intracellular cholesterol transporter 2**	− **0.27**	− **0.29**	− **0.53**	− **0.49**
Q61171	Prdx2	Peroxiredoxin-2	-	− 0.54	-	-
Q9Z282	Gpr132	Probable G-protein coupled receptor 132	0.48	0.74	-	-
O08976	Pbsn	Probasin	-	-	0.83	-
Q9ESD1	Prss8	Prostasin	-	-	-	− 0.61
Q3UN54	Pate14	Prostate and testis expressed protein 14	0.32	-	-	-
Q09098	Pate4	Prostate and testis expressed protein 4	-	0.40	-	-
Q9D309	Fam3b	Protein FAM3B	0.41	0.44	0.43	-
P41438	Slc19a1	Reduced folate transporter	-	-	-	− 0.24
Q8K1J5	Sde2	Replication stress response regulator SDE2	-	-	− 0.42	-
P18419	Svs4	Seminal vesicle secretory protein 4	-	0.26	0.48	0.26
P30933	Svs5	Seminal vesicle secretory protein 5	-	0.73	-	-
Q64356	Svs6	Seminal vesicle secretory protein 6	-	0.57	-	0.38
Q8CEK3	Spinkl	Serine protease inhibitor kazal-like protein, minor form	-	-	-	− 0.39
P09036	Spink1	Serine protease inhibitor Kazal-type 1	0.36	-	-	− 0.45
Q921I1	Tf	Serotransferrin	-	− 0.53	-	-
O88968	Tcn2	Transcobalamin-2	-	-	0.86	0.45
Q5U405	Tmprss1	Transmembrane protease serine 13	-	0.51	0.76	-
Q8K0J2	B3gnt7	UDP-GlcNAc:betaGal beta-1,3-N-acetylglucosaminyltransferase 7	-	-	− 0.28	− 0.37
P06869	Plau	Urokinase-type plasminogen activator	-	0.39	0.45	-

Seminal vesicle fluid proteins were assessed using the *R*-package Limma via online StatsPro software to determine significantly differentially expressed proteins (*padj* < 0.05). Numbers demonstrate either up or down log2Fold Change, with - indicating no detectable change. Proteins highlighted in bold indicate common changes between all comparison groups. Seminal vesicle fluid extracted from *n* = 6 males per group

UniProt tissue-specific annotations of the differentially expressed proteins (DEPs) from seminal vesicle fluid demonstrated that all detected DEPs have key involvements in various reproductive tissues, as they are located in the testis and accessory glands (Fig. 7C). LPD demonstrated the most DEPs associated with the prostate (27.3%), whereas WD was found to have similar proportions of DEPs associated with both the prostate and testes (27.3% of DEPs in each location). Methyl donor supplemented groups (MD-LPD and MD-WD) revealed the most DEPs associated with the testis (31.6% and 45.5% respectively) (Fig. 7C). Whilst this highlights these DEPs and key reproductive associated proteins, it must be noted that the proteins are not exclusive to these tissue locations. The majority of seminal vesicle fluid DEPs were found to be secretory proteins in all dietary groups except MD-WD, which had equal secretory and cell membrane associated DEPs (Fig. 7D). Interestingly, in LPD groups (LPD and MD-LPD), the methyl donor supplementations increased the number of secreted DEPs found in seminal vesicle fluid, whereas in WD groups (WD and MD-WD), this was the opposite, with methyl donor supplementations reducing the number of secreted DEPs (Fig. 7D). The biological processes associated with the differentially abundant proteins in each group were examined using DAVID web-based software; there was minimum overlap with the processes found to be affected by the protein changes in response to sub-optimal diet (Table 2). Response to LPD and MD-LPD had no common biological process influences, whereas WD and MD-WD both showed common changes to the modulation of age-related behavioural decline (GO:0090647) and the response to cadmium ion (GO:0046686), both due to changes in beta-2-microglobulin and major prion protein. Both methyl donor containing diets (MD-LPD and MD-WD) had seminal fluid protein changes that suggest an alteration in proteolysis pathways (GO:0006508).

**Table 2:** Gene ontology analysis of the significantly dysregulated seminal vesicle fluid proteins identifying implicated biological processes

Comparison	Biological process	GO term	# proteins	FDR
**CD vs LPD**	Negative regulation of peptidase activity	GO:0010466	3	0.16
	Cell activation	GO:0001775	2	0.33
	Intermediate filament organisation	GO:0045109	2	0.97
**CD vs MD-LPD**	Proteolysis	GO:0006508	6	0.08
	Regulation of smooth muscle cell migration	GO:0014910	2	0.61
	Negative regulation of plasminogen activation	GO:0010757	2	0.61
	Metabolic process	GO:0008152	3	0.61
	Peptide metabolic process	GO:0006518	2	0.61
	Response to lipopolysaccharide	GO:0032496	3	0.61
	Negative regulation of protein processing	GO:0010955	2	0.61
	Regulation of immune system process	GO:0002682	2	0.61
	Carbohydrate metabolic process	GO:0005975	3	0.61
	Negative regulation of proteolysis	GO:0045861	2	0.99
**CD vs WD**	Learning or memory	GO:0007611	3	0.56
	Modulation of age-related behavioural decline	GO:0090647	2	0.64
	Metabolic process	GO:0008152	3	0.64
	Response to hypoxia	GO:0001666	3	0.64
	Carbohydrate metabolic process	GO:0005975	3	0.64
	Regulation of immune system process	GO:0002682	2	0.64
	Positive regulation of receptor-mediated endocytosis	GO:0048260	2	0.72
	Response to cadmium ion	GO:0046686	2	0.78
	Acute-phase response	GO:0006953	2	0.81
**CD vs MD-WD**	Negative regulation of calcium ion import	GO:0090281	2	0.59
	Modulation of age-related behavioural decline	GO:0090647	2	0.59
	Proteolysis	GO:0006508	4	0.59
	Positive regulation of protein tyrosine kinase activity	GO:0061098	2	0.92
	Response to cadmium ion	GO:0046686	2	0.92

GO Terms were generated by using free-web based software, DAVID Bioinformatics Resources, with all significantly differentially expressed (*padj* < 0.05) seminal vesicle fluid proteins from each comparison used as input lists. Seminal vesicle fluid extracted from *n* = 6 males per group

### Paternal Western diet alters preimplantation uterine immune and inflammatory marker gene expression

**Fig. 8:**
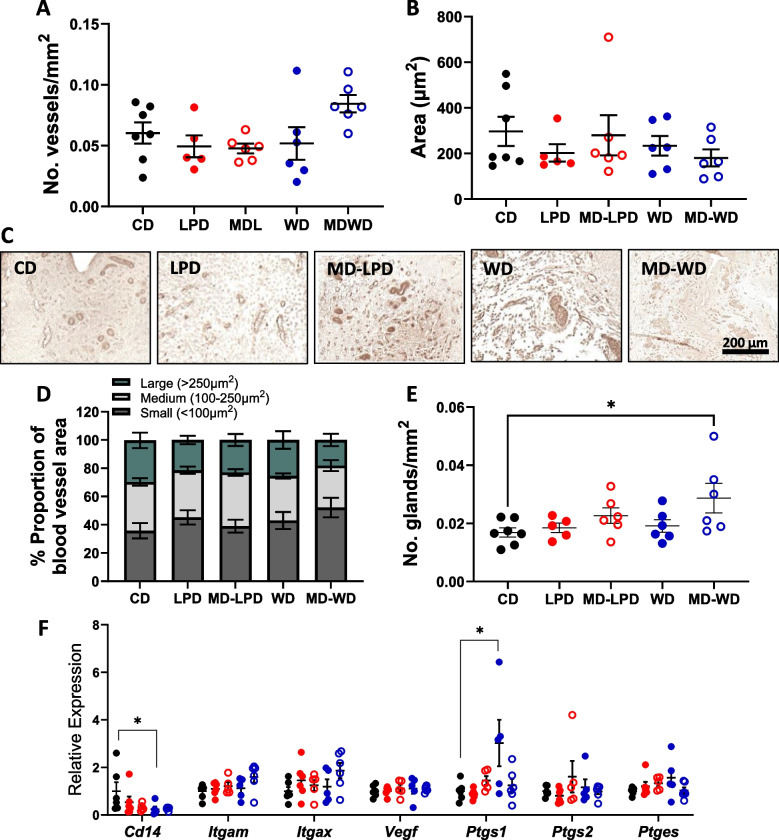
Maternal uterine physiology is altered in response to paternal diet. **A** The number of CD31^+^ blood vessels per mm^2^ of uterine tissue and (**B**) the average area of blood vessels across whole uterine Sect. (100 vessels measured per section). **C** Representative images of CD31^+^ blood vessels in the uterus on day 3.5 of gestation from females mated with CD, LPD, MD-LPD, WD and MD-WD males. **D** The proportional distribution of blood vessel sizes from imaged tissue, categorised into small (< 100 μm^2^), medium (100–250 μm^2^) or large (> 250 μm^2^) area vessels. **E** The number of uterine glands per mm.^2^ of uterine tissue (CD *n* = 7, LPD *n* = 5, MD-LPD *n* = 6, WD *n* = 6, MD-WD *n* = 6). **F** Whole uterus gene expression of key immune regulatory and inflammatory genes presented relative to CD expression, normalised using GeNorm method against *Tbp* and *Ppiβ*. Data points represent average for one dam (CD *n* = 7, LPD *n* = 7, MD-LPD *n* = 7, WD *n* = 7, MD-WD *n* = 7) with average ± SEM. Statistical significance determined using one-way ANOVA with Tukey’s post-test; *p < 0.05

As we observed seminal fluid composition changes, and previous studies have found changes to paternal diet impacts maternal uterine vasculature, we examined the early (embryonic day (E)3.5) uterine vascular morphology in response to mating with intact CD, LPD, MD-LPD, WD and MD-WD males. We observed no difference in the number of uterine blood vessels per mm^2^ of the uterine luminal epithelium (Fig. 8A), or in the mean vessel cross-section area (Fig. 8B), as determined using immunohistochemistry for platelet endothelial cell adhesion molecule (CD31) (Fig. 8C). Proportionally, females mated to CD males demonstrated a relatively equal distribution of small, medium and large vessel areas in the uterine tissue examined (a ratio of 1:1:0.8 respectively) (Fig. 8C). Whilst uteri mated with LPD, MD-LPD and WD males did not deviate greatly from this pattern, there was a slight (*p* = 0.1034) increase in the proportion of small area vessels in females mated to MD-WD-fed males compared to those mated to CD-fed males (52 ± 6.9% vs 36 ± 5.4%). However, MD-WD females displayed a greater number of uterine glands when compared to CD females (Fig. 8E; *p* = 0.023).

We also examined the expression profiles of several genes involved in immunological responses (*Cd14*,* Itgam*,* Itgax*) and angiogenesis and inflammation (*Vegf*,* Ptgs1*,* Ptgs2*,* Ptges*) within the same uterine tissue (contralateral horn to that used for immunohistochemistry). Expression of *Cd14* was significantly decreased whilst *Ptgs1* was increased in uteri from dams mated to WD-fed males (Fig. 8F; *p* < 0.05) when compared to uteri from dams mated to CD-fed males.

## Discussion

The relevance of paternal diet in relation to offspring outcome and future health is becoming more prominent in reproductive research. However, the mechanisms by which these changes are brought about in utero are still not fully understood. Our current study shows that both paternal undernutrition (LPD) and overnutrition (WD) affect a continuum of metabolic and reproductive traits in males which, ultimately influence embryonic development and the post-mating maternal environment. We observe that LPD had minimal impact on male lipid metabolism whilst WD changed the abundance of over 350 lipids. Similarly, in the sperm, LPD altered the expression of 27 mRNAs, whilst in WD sperm, there were 82 differentially expressed transcripts. However, both LPD and WD increased the developmental kinetics of preimplantation embryos, with an enhanced rate of development evident from the earliest stages. Interestingly, supplementation of our LPD and WD with methyl donors had differential affects, dependent on the base diet supplemented. These observations suggest that a father’s nutritional status at the time of conception is critical for directing his own reproductive health and the well-being of his offspring.

We observed minimal differences in the growth profiles in our males fed either the LPD or WD (with or without methyl donors). This observation contrasts with other studies that showed an increased weight gain in mice fed high-fat/high-sugar diets [31–34]. However, these studies typically used diets that had higher energy content from fats [33] or were supplemented with additional forms of sugar (such as increased fructose in the drinking water [32] or ad libitum access to sweetened condensed milk [31]) which could somewhat explain our lack of WD-associated weight gain. However, at cull, the WD males did display an increased adiposity, specifically in the white adipose deposits in the gonadal region, yet showed a decreased body weight and reduced brown adipose tissue (interscapular adipose) mass, suggesting a loss of some lean-mass. Obesity and high-fat/calorie diets in humans and rodents have been associated with anabolic resistance at both muscle and whole-body levels [35, 36]. Increased obesity has also been associated with muscle accumulation of toxic fatty acids and derivatives such as ceramides resulting in lipotoxicity in rats [37]. This is driven, in part, through excess circulatory lipids being stored in non-adipose tissues such as muscle and the liver. This accumulation of toxic lipid derivatives in the muscle then results in decreased anabolic profiles as well as reductions in muscle strength and poor physical performance in both animals and humans [38, 39]. Indeed, WD- and MD-WD-fed males displayed elevated liver weights and increased gonadal fat weight suggesting increased fat storage compared to all other diet groups. These observations are supported further by the dramatic changes in serum lipid profiles seen in both WD and MD-WD males. Typically, in animal studies, methyl donor supplementation has been associated with beneficial reductions in weight and lipid metabolism [40, 41]. However, some studies have shown that deficiencies in folate, methionine and choline decrease lipid metabolism through the phosphatidylethanolamine *N-*methyltransferase (PEMT) pathway [42]. Furthermore, a high-fat diet in combination with elevated folate intake has been shown to result in hepatic lipid accumulation and impaired fatty acid oxidation in rats [43]. Interestingly, the expression of genes and proteins involved in fatty acid metabolism, such as Elovl2 and Fads2, are regulated through their DNA methylation status [44, 45]; however, we did not observe definitive increases the methyl donor-associated lipid profiles in LPD compared to MD-LPD or WD compared to MD-WD. The most notable differences in serum lipid profiles in this study were driven by the male exposure to a WD, irrespective of methyl donor supplementation. Whilst the lipid profiles of MD-WD and WD males were not identical, both were associated with a vast increase in lipid number when compared to CD, whereas LPD and MD-LPD comparison groups showed minimal changes. This dysregulation of serum lipids in diets with high fat, sugar and cholesterol has been observed in other models of high-fat feeding in rodents and humans and is indicative of dyslipidaemia [46–48].

Whilst the WD and MD-WD males displayed significant changes in their central metabolic profile, we observed minimal differences in testicular morphology or abundance of 1-carbon metabolites. However, analysis of sperm mRNA content identified a range of differentially expressed transcripts in all experimental groups. Sperm RNA populations in men have been found to be heterogeneous; thus, a conclusion regarding the importance or significance of certain mRNA in terms of infertility markers is still much discussed. However, there is evidence in humans that sperm RNA populations are acutely responsive to changes in diet, such as increases in sugar intake [49]. In mice, male obesity perturbs the abundance of multiple classes of RNAs, including mRNA, miRNA and tRNA [24,50,51]. In our LPD and MD-LPD groups, we observed an overall decreased in transcript abundance when compared to CD sperm. Similarly, in WD and MD-WD sperm, the majority of differentially expressed transcripts were decreased relative to CD sperm. Only 6 transcripts (*Gm43064*, *Apoa1*, *Pcdhga10*, *Apoa4*, *Gc* and *Fgg*) were differentially expressed in all groups. Such common changes in sperm mRNA content could occur as a consequence of impaired testicular function and spermatogenesis [52, 53]. For example, impaired vitamin D metabolism, mediated in part through the vitamin D receptor (*Gc*), has been linked to increases in reactive oxygen species levels [54] and perturbed testosterone production [55]. Similarly, expression of apolipoprotein A1 (*Apoa1*) is critical for the transport of cholesterol, upon which the synthesis of testosterone is dependent. Furthermore, the observation that all groups displayed differential expression of *Gm43064*, an alternative splice variant of *Ap4b1*, could indicate perturbed patterns of testicular transcription. Therefore, the changes identified here could be a marker of a perturbed testicular environment and impaired spermatogenesis.

Sperm RNA populations have also been identified as potential regulators of early embryonic development [56]. Dynamic changes to sperm RNA load have been observed as they transit through the caput and caudal epididymis, and these RNAs are delivered to the zygote and can influence embryo gene expression as early as the 4-cell stage [57]. Similarly, a study investigating small RNA populations in mice found that high-fat diet alters the RNA sperm profile and that these RNAs influence early embryo expression of genes involved in metabolic regulation [24]. As sperm contain just 1% of a somatic cell’s RNA profile [58], altering the transcriptional landscape of the early embryo prior to zygotic genome activation allows for the small quantity of sperm RNA to maximise their influence on future development and offspring fitness. This early impact on pre-implantation embryo development may be one mechanistic route for paternal-driven developmental programming observed in multiple studies examining poor paternal diet and offspring health [18, 25, 59, 60].

Several studies have found that an insult to father’s sperm or paternal sub-optimal environment leads to delayed preimplantation embryo development [61, 62]. Damage to sperm, such as DNA fragmentation and oxidative stress damage, has been found to reduce development rate leading to embryos taking longer to reach the blastocyst stage and fewer achieving blastulation [63]. Obese mice have also been found to produce embryos that have delayed preimplantation development [18]. Binder et al. utilised in vitro fertilisation to produce embryos from sperm taken from obese stud males, leading to significant developmental delays in vitro. However, a study examining increased paternal BMI in humans found an accelerated rate of embryo development, similar to our current study. Here, a higher paternal BMI preconception was associated with a faster preimplantation development rate up to the 8-cell stage, which correlated to a reduced fertilisation potential [64]. Separately, studies examining the influence of sperm from infertile men have also identified increased rates of early preimplantation embryo development [65, 66]. These studies suggest male infertility is associated with a lower level of sperm chromatin condensation, allowing faster access of the oocyte’s remodelling machinery to the paternal genome. This hypothesis is supported by observations of increased embryo developmental rates when using immature testicular sperm versus mature epidermal sperm in human assisted reproduction [67]. However, a quicker transition through the first cell cycle in the preimplantation embryo could reduce the time allowed for DNA damage repair, enabling paternal mutations to become established within the offspring’s DNA [68]. Interestingly, we found that methyl donor supplementation did not shift the embryos developmental trajectory towards that of embryos from the control group. Suggesting that the typical ‘poor’ diets (LPD and WD) cannot be simply corrected by dietary supplementation with vitamins and minerals that influence methylation status, the methylation status of the sperm cannot be solely responsible for the altered morphokinetics of the early embryo observed in the present study.

Whilst poor paternal diet and lifestyle have been connected with changes in sperm quality and epigenetic status, there is increasing evidence that the seminal plasma can also influence embryonic, foetal and postnatal offspring wellbeing [26]. Previously, we have shown that seminal plasma from LPD-fed male mice programmed poor metabolic and cardiovascular health in the offspring for up to two generations [29, 30, 69]. Separately, absence of seminal plasma at the time of mating in mice impaired embryo development and programmed poor cardio-metabolic health in the offspring [70]. In our current study, we observed paternal diet altered the relative abundance of multiple proteins within the seminal vesicle fluid, albeit only minor changes were detected with the majority of proteins demonstrating only minor fold changes. Of the observed changes, however, only two proteins, multiple inositol polyphosphate phosphatase 1 and NPC intracellular cholesterol transporter 2, were found to be differentially abundant in all groups (up and down regulated respectively). Changes in such proteins might reflect metabolic changes shared across all the males and could act as a biomarker of general male reproductive health. Indeed, in some species, seminal plasma composition has been identified as a marker of male reproductive fitness [71]. Separately, in species such as mice, and humans to a lesser extent, seminal plasma plays a significant role in modulating the maternal reproductive tract following insemination [72]. Various bioactive factors, such as cytokines, nucleic acid packages and several hormones are present in the seminal fluid of males. We found in this study that females mated to WD-fed males had dysregulated uterine expression of *Cd14* and *Ptgs1*, suggesting uterine immunological responses to paternal semen factors could be altered by paternal diet. Prostaglandins have a key role in implantation, particularly in embryo development, decidualisation, angiogenesis and immune cell infiltration, all key processes that can be directly and indirectly altered by male factors [26, 73]. Prostaglandin synthase 1 (*Ptgs1*) is constitutively expressed in the uterus during the preimplantation period and is not as susceptible to inducement as its counterpart, prostaglandin synthase 2 (*Ptgs2*). However, we observed a significant reduction in uterine *Ptgs1* expression in response to mating with WD-fed males. PTGS1 (along with PTGS2[74–76]. With this gene expression reduced in females mated to WD-fed males, it suggests a potential dysregulation in the prostaglandins present in the uterus. However, as there were no alterations in the more inducible *Ptgs2* gene, this suggests a more detailed profiling of the prostaglandin presence and synthesis in uterine tissue exposed to nutritionally altered seminal fluid is required. Interestingly, Watkins et al. have previously demonstrated paternal LPD significantly reduced uterine expression of prostaglandin synthesis genes, *Alox5*, *Ptgs2*, *Ptges* and *Tbxas1*, as well as reduced the overall endometrial [20]. However, we did not find any evidence of sub-optimal paternal diets altering uterine vessel area, which could be attributed to the lack of observed changes to angiogenesis-associated genes. *Vegf* [77, 78] gene expression was also detected in uterine tissue at E3.5, representing the monocyte, macrophage and dendritic cell populations, respectively [77, 78]. In females mated to WD-fed males, uterine expression of the monocyte cell marker, *Cd14*, was significantly decreased, suggesting that a paternal high-fat, high-sugar diet may alter seminal immunomodulatory cytokines, potentially bringing about a reduced infiltration of monocytes within the decidua.

## Conclusions

This study supports previous evidence that a paternal diet does play a role in regulating early preimplantation development in the mouse. In summary, we have shown that both LPD and WD-fed male mice sire embryos have an accelerated preimplantation development rate and that a poor diet supplemented with methyl donors does not correct this altered development trajectory. Our data indicate that poor paternal diet alters the sperm RNA landscape and the composition of the seminal vesicle fluid. We propose that perturbations in these seminal constituents direct embryo developmental dynamics both directly (sperm mediated) and indirectly (seminal plasma mediated), shaping the long-term health of the resultant offspring.

## Methods

### Mice diet regime, matings and tissue collection

All animal procedures were approved by the UK Home Office according to the Animals (Scientific Procedures) Act 1986, Amendment Regulations 2012, and carried out under Project License 30/3253 with local ethical approval at University of Nottingham. C57BL/6 J mice (Charles River, UK) were housed in controlled 12/12-h light/dark conditions with a constant temperature (21 °C ± 3 °C) and ad libitum access to water. Virgin 8-week old males were fed either control diet (CD; 18% casein, 21% sugar, 0% milk fat, 0% cholesterol [0.74 kcal/g protein, 0.56 kcal/g fat, 1.80 kcal/g carbohydrates], *n* = 28), isocaloric low-protein diet (LPD; 9% casein, 24% sugar, 0% milk fat, 0% cholesterol [0.37 kcal/g protein, 0.56 kcal/g fat, 2.17 kcal/g carbohydrates], *n* = 30), ‘Western’ diet (WD; 19% casein, 34% sugar, 20% milk fat, 0.15% cholesterol [0.70 kcal/g protein, 1.93 kcal/g fat, 2.00 kcal/g carbohydrates], *n* = 30) or LPD or WD supplemented with methyl donors [an addition of 5 g/kg diet choline chloride, 15 g/kg diet betaine, 7.5 g/kg diet methionine, 15 mg/kg diet folic acid, 1.5 mg/kg diet vitamin B_12_ to either LPD (MD-LPD, *n* = 29) or WD (MD-WD, *n* = 28)] for a minimum of 8 weeks. Exact dietary formulations are outlined in Additional file 8: Table S7.

Virgin female C57BL/6 J mice were mated at 9-weeks old (± 7 days) with stud males from one of the 5 diet groups. Successful mating was confirmed by the presence of a copulation plug, denoted as embryonic day (E) 0.5. After successful mating, the males remained on their respective diets until they were euthanized and sperm retrieved from the caudal epididymis. Briefly, both epididymides were roughly sliced in warmed M2 media (M7167- Sigma Aldrich) and were left for 30 min at 37° C for the sperm to swim up into fresh media. The total live sperm fraction was snap frozen for RNA extraction. Seminal vesicles were excised and fluid obtained for proteomics. The liver, testis and adipose tissue were weighed and collected for further analysis, and blood serum was processed as described below. The dams were fed and maintained on standard rodent chow (rat/mouse no.1 maintenance diet, Special Diet Services) and euthanized via cervical dislocation on E1.5 for embryo collection (CD; *n* = 5, LPD; *n* = 6, MD-LPD; *n* = 6, WD; *n* = 7, MD-WD; *n* = 6) or E3.5 for pre-implantation uterine tissue collection (CD; *n* = 7, LPD; *n* = 7, MD-LPD; *n* = 7, WD; *n* = 7, MD-WD; *n* = 7).

### Serum untargeted lipidomics

#### Sample preparation

Male serum (*n* = 8/diet group) was prepared by placing blood on ice to allow coagulation and then centrifuged at 4 °C for 10 min at 10,000 × g. Serum samples were extracted to separate low molecular weight metabolites from other biochemicals including proteins, RNA and DNA. Samples were thawed and extracted on ice. For the analysis of water-soluble metabolites, 150 μL of acetonitrile/methanol (1:1 (v/v), LC–MS grade, LiChrosolv, Merck) was added to 50 μL of plasma followed by vortex mixing (20 s), centrifugation (22,000 × g, 20 min at 4 °C) and transfer of the clear supernatant to a glass LC autosampler vial (VI-04–12-02RVG 300μL Plastic, Chromatography Direct, UK). For the analysis of lipid metabolites, 150 μL of isopropyl alcohol, IPA (LC–MS grade, LiChrosolv, Merck), was added to 50 μL of plasma followed by vortex mixing (15 s), centrifugation (22,000 × g, 20 min at 4 °C) and transfer of the clear supernatant to a glass LC autosampler vial (VI-04–12-02RVG 300 μL Plastic, Chromatography Direct, UK). A single pooled QC sample was prepared by combining aliquots of all biological samples where adequate sample volume remained and vortex mixing (2 min). Aliquots (50 μL) of the pooled QC sample were extracted as defined above.

#### Ultra-performance liquid chromatography-mass spectrometry

Samples were analysed applying two ultra-performance liquid chromatography-mass spectrometry (UPLC-MS) methods using a Dionex UltiMate 3000 Rapid Separation LC system (Thermo Fisher Scientific, MA, USA) coupled with a heated electrospray Q Exactive Focus mass spectrometer (Thermo Fisher Scientific, MA, USA). Polar extracts were analysed on a Accucore-150-Amide-HILIC column (100 × 2.1 mm, 2.6 μm, Thermo Fisher Scientific, MA, USA). In positive ion mode UPLC, solvents consisted of mobile phase A which consisted of 10 mM ammonium formate and 0.1% formic acid in 95% acetonitrile/water and mobile phase B which consisted of 10 mM ammonium formate and 0.1% formic acid in 50% acetonitrile/water. In negative ion mode UPLC, reservoir solvents consisted of 10 mM ammonium acetate and 0.1% acetic acid in 95% acetonitrile/water, and mobile phase B consisted of 10 mM ammonium acetate and 0.1% acetic acid in 50% acetonitrile/water. Flow rate was set for 0.50 mL·min^−1^ with the following gradient: *t* = 0.0, 1% B; *t* = 1.0, 1% B; *t* = 3.0, 15% B; *t* = 6.0, 50% B; *t* = 9.0, 95% B; *t* = 10.0, 95% B; *t* = 10.5, 1% B; *t* = 14.0, 1% B; all changes were linear with curve = 5. The column temperature was set to 35 °C, and the injection volume was 2 μL. Data were acquired in positive and negative ionisation modes separately within the mass range of 70–1050 m/z at resolution 70,000 (FWHM at m/z 200). Ion source parameters were set as follows: sheath gas = 55 arbitrary units, aux gas = 14 arbitrary units, sweep gas = 4 arbitrary units, spray voltage = + 3.5 kV/ − 2.7 kV, capillary temp. = 380 °C, aux gas heater temp. = 440 °C.

Non-polar extracts were analysed on a Hypersil GOLD column (100 × 2.1 mm, 1.9 μm; Thermo Fisher Scientific, MA, USA). Mobile phase A consisted of 10 mM ammonium formate and 0.1% formic acid in 60% acetonitrile/water, and mobile phase B consisted of 10 mM ammonium formate and 0.1% formic acid in 90% propan-2-ol/water. Flow rate was set for 0.40 mL·min^−1^ with the following gradient: *t* = 0.0, 20% B; *t* = 1.6, 20% B, *t* = 9.4, 100% B; *t* = 10.6, 100% B; *t* = 12.6, 20% B; *t* = 15.0, 20% B; all changes were linear with curve = 5. The column temperature was set to 55 °C, and the injection volume was 2 μL. Data were acquired in positive and negative ionisation mode separately within the mass range of 150–2000 m/z at resolution 70,000 (FWHM at m/z 200). Ion source parameters were set as follows: sheath gas = 48 arbitrary units, aux gas = 15 arbitrary units, sweep gas = 0 arbitrary units, spray voltage = + 3.5 kV/ − 2.7 kV, capillary temp. = 350 °C, aux gas heater temp. = 400 °C.

A Thermo ExactiveTune 2.8 SP1 build 2806 was used as instrument control software in both cases, and data were acquired in profile mode. Quality control (QC) samples were analysed as the first ten injections and then every seventh injection with two QC samples at the end of the analytical batch. Two blank samples were analysed, the first as the 6th injection and then the second at the end of each batch.

#### Raw data processing

Raw data acquired in each analytical batch were converted from the instrument-specific format to the mzML file format by applying the open access ProteoWizard software [79]. Deconvolution was performed with XCMS software [80] according to the following settings: min peak width (4 for HILIC and 6 for lipids), max peak width [30], ppm (12 for HILIC and 14 for lipids), mzdiff (0.001), gapInit (0.5 for HILIC and 0.4 for lipids), gapExtend (2.4), bw (0.25), mzwid (0.01). A data matrix of metabolite features (m/z-retention time pairs) vs. samples was constructed with peak areas provided where the metabolite feature was detected for each sample.

#### Metabolite annotation

Putative metabolite annotation was performed applying the Python package BEAMSpy (RT diff = 2 s, Pearson correlation > 0.70, *p-*value < 0.05); m/z values of all experimentally observed peaks were searched against HMDB [81] and LIPIDMAPS [82], and all matches within a 5-ppm mass error tolerance were reported. All metabolites described are reported to level 2 or 3 as defined by the Metabolomics Standards Initiative [83]. Comparisons for all sub-optimal diet groups (LPD, MD-LPD, WD, MD-WD) were made against control diet (CD).

#### Quality control and quality assessment

A quality assurance and quality control (QA/QC) assessment was performed to measure drift across retention time, m/z and signal intensity and identify potential outliers. The first five QCs were used to equilibrate the analytical system and therefore subsequently removed from the data before the data was analysed. Principal component analysis (PCA) was performed to assess the technical variability (measured by the replicate analysis of a pooled QC sample) and biological variability as part of the quality control process. Prior to PCA, missing values in the data were replaced by applying k-nearest neighbour (kNN) missing value imputation (*k* = 5) followed by probabilistic quotient normalisation (PQN) [84] and glog transformation [85] prior to data analysis. The data from the pooled QC samples were applied to perform QC filtering. For each metabolite feature detected, the relative standard deviation and percentage detection rate were calculated using the remaining QC samples. Blank samples at the start and end of a run were used to remove features from non-biological origins. Any feature with an average QC intensity less than 20 times the average intensity of the blanks was removed. Any sample with > 50% missing values was excluded from further analysis. Metabolite features with a RSD > 30% and present in less than 90% of the QC samples were deleted from the dataset. Features with a < 50% detection rate over all samples were also removed.

#### Statistical analysis

Multivariate statistical analysis.

The multivariate analysis was carried out in Matlab (The MathWorks, Inc., Natick, MA, USA) using scripts based on functionality provided by the PLS_Toolbox (Eigenvector Research, Inc., Manson, WA USA 98831). Prior to the statistical analysis, probabilistic quotient normalisation (PQN), using the mean of the QC samples as a reference, was applied. k-nearest neighbour missing value imputation (*k* = 5) was used to replace missing values, and a glog-transformation (mean QC applied as a reference) of the data was performed. Partial least squares (PLS) discriminant analysis (DA) was used to determine significant features. The number of latent variables to use in the PLS model was established using a grid search over 1 to 15 components inclusive, using a permuted (10 repetitions), k-fold cross-validation model to determine the number of components that minimises the average test set prediction error over all classes. The number of folds *k* is set equal to the sqrt (number of samples) for a given matrix. Variable importance for protection (VIP) scores for each feature were calculated using PLSDA applied to the full sample set using the optimum number of components. Permutation tests (50 repetitions) were used to establish the validity of the model (*p* < 0.05). Forward selection was used to identify the number of peaks with highest VIP scores as significant.

### Liver and testis targeted metabolomics

#### Materials

All mobile phases were prepared using LC–MS grade solvents. Formic acid was purchased from Sigma-Aldrich. Water, acetonitrile and methanol were purchased from VWR International. Methionine, pyridoxine, vitamin B_12_, homocysteine, S-adenosylhomocysteine (SAH), methionine-(methyl-13C,d3) and folic acid-(glutamic acid-13C5,15N) were purchased from Sigma Aldrich. Homocysteine-3,3,4,4-d4 and pyridoxine-d2 HCl (5-hydroxymethyl-d2) were purchased from CDN Isotopes. S-(5′-adenosyl)-L-methionine (tosylate) (SAM), 5-methyltetrahydrofolic acid (5MTHF), folic acid and S-adeonosylhomocysteine-d4 (SAH-d4) were purchased from Cayman Chemical.

#### Sample preparation

Liver and testis (*n* = 8/diet group) tissue samples and calibration/QC samples were prepared using a biphasic extraction method (Bligh and Dyer). Weighed tissue samples were transferred to CK14 Precellys tubes, and 16 μL/mg ice-cold methanol and 5.7 μL/mg ice-cold water were added to each tube and homogenised (2 × 10 s bursts at 6400 rpm, room temperature) using the Precellys24 system (Bertin Instruments, Stretton, UK). Once homogenised, the samples were immediately placed on ice. For calibration samples, the tissue was diluted 1:500 with ice-cold 73.7/26.3 methanol/water (v/v). For study samples, all homogenates were analysed twice: [1] using 30–40 mg for testes samples and 70 mg for liver samples, and [2] all homogenates were diluted 1:10 (required for the quantification of methionine, SAH and SAM). The appropriate volume of homogenate was transferred to glass vials containing 16 μL/mg chloroform (320 μL for calibration, QC samples and 1:10 diluted study samples which is the equivalent volume for 20 mg) and 8 μL/mg water (120 μL of the appropriate calibrant and 40 μL ISTD for calibration and QC samples). Forty microliters of ISTD was added to all samples at this stage. The glass vials were vortexed, incubated on ice for 10 min and centrifuged at 2500 g at 4 °C for 10 min followed by incubation at room temperature for 5 min. The polar phase (top phase) was then dried down using a speed vac and reconstituted in 50 μL of 99/1 water/methanol (v/v) and analysed. All calculated concentrations were then corrected for the appropriate concentration dilution factor.

#### Liquid chromatography and mass spectrometry conditions

Targeted quantification of key metabolites of the central one-carbon metabolism pathway (methionine, S-adenosylmethionine, S-adenosylhomocysteine and homocysteine) was performed using a TSQ Quantiva triple quadrupole mass spectrometer (Thermo Scientific, Bremen) coupled to an Ultimate 3000 UHPLC (Thermo Scientific, Bremen). The LC separation was performed using an Acquity UPLC® HSS T3 column (1.8 μm, 150 × 2.1 mm, Waters Ltd) operated at a temperature of 45 °C. Solvents A and B consisted of 99.9% water/0.1% formic acid (A) and 99.9% methanol/0.1% formic acid (B). An injection volume of 3 μL and a flow rate of 0.3 mL·min^−1^ were applied. Separation applied a gradient elution as follows: 0–1 min, 1%B (curve 5); 1–8 min, gradient to 99%B (curve 7); 8–10 min, 99%B (curve 5); 10–12 min, gradient to 1%B (curve 3); 12–15 min, 1%B (curve 5). Detection was performed in positive ion mode using the following source parameters: positive ion spray voltage, 2000; sheath gas, 35; aux gas, 10; sweep gas, 0; ion transfer tube temperature, 325 °C; vaporiser temperature, 300 °C. Needle wash was 50% acetonitrile, and the rear seal wash was 10% methanol. Data acquisition was performed using Xcalibur 4.0, and raw data processing was performed using TraceFinder 4.1 (Thermo Scientific, San Jose).

#### Multiple reaction monitoring (MRM) optimisation

Multiple reaction monitoring (MRM) optimisation was performed using the automated function in TSQ Quantiva Tune 2.0 by direct infusion of individual standards (25–50 μg·mL^−1^). The optimised collision energy and RF lens voltage for the optimised MRM transitions for each analyte are shown in Additional file 9: Table S8.

#### Calibration curve and internal standard (ISTD) preparation

The highest calibration point was prepared in water containing standards at the following concentrations: methionine, 1.5 μg/ml; pyridoxine, 250 ng/ml; SAH, 14 μg/ml; SAM, 6 μg/ml; folic acid, 6 μg/ml; 5MTHF, 15 μg/ml; vitamin B_12_, 2.5 μg/ml; homocysteine, 2.5 μg/ml. The calibration curve was then prepared using a 1-in-2 serial dilution method. The final concentrations were expressed as pg/mg for all analytes except SAM (pmoles/mg).

The ISTD was prepared in water containing labelled standards at the following concentrations: methionine-(methyl 13C3) d3, 250 ng/ml; pyridoxine-d2, 250 ng/ml; SAH-d4, 5 μg/ml; folic acid-(glutamic acid-13C5,15N), 5 μg/ml; homocysteine-d4, 3.75 μg/ml.

During assay validation, the linear range for each standard was as follows: methionine, 17.6–4500 pg/mg; pyridoxine, 5.9–3000 pg/mg; SAH, 82–84,000 pg/mg; folic acid, 140.6–9000 pg/mg; homocysteine, 117.2–15,000 pg/mg; 5MTHF, 70.31–22,550 pg/mg; SAM, 0.123–63.084 pmol/mg; vitamin B_12_ 14.6–3750 pg/mg. Linearity, LLOQ, ULOQ, precision and accuracy was assessed to the validation guidelines criteria recommended by U.S. Department of Health and Human Services, Food and Drug Administration (FDA).

#### Statistical analysis

Univariate statistical analysis.

All univariate statistics were performed in the R environment using the Univariate statistics function made available by Workflow4Metabolomics. Probabilistic quotient normalisation (PQN, mean QC applied) of the data was performed prior to the application of ANOVA (analysis of variance); *p* < 0.05 was used to identify features showing a significant difference in intensities between groups. This requires the application of multiple tests (one for each metabolite feature), so a false discovery rate (FDR) correction was performed to control the false discovery rate. Post hoc (Tukey, *p* < 0.05) was applied to significant features in order to determine in which groups a feature is elevated/reduced.

### Testis histology

Formalin fixed, paraffin-embedded whole testis (*n* = 8/diet group) were sectioned to 5 μm and stained for morphological analysis using standard H&E stain. Stained sections were imaged by a blinded operator at × 20 magnification using Leica DMRB microscope with an Oasis GlideScanner and Surveyor software to stich individual images to create of whole testis cross-sectional area image file. An average of 50 seminiferous tubule cross-section areas were measured for each of the testis samples using the ImageJ software.

### Sperm RNA sequencing

Sperm was isolated (*n* = 6/diet group), as described in the earlier section pertaining to tissue collection, and RNA was extracted using Qiagen miRNeasy micro kit following manufacturer’s instructions with homogenisation in Qiazol using TissueLyser II. Total RNA sequencing libraries were prepared using NEBNext Ultra II Directional RNA Library Preparation Kit for Illumina (NEB; E7760) and NEBNext Multiplex Oligos for Illumina (96 Unique Dual Index Pairs) (NEBNext; E6440). Libraries were pooled and sequenced on the Illumina NextSeq500, to generate over 40 million pairs of 75-bp paired-end reads per sample. Reads were trimmed to remove nucleotides of quality score < 20 using TrimGalore (v0.6.7) [86] and aligned to reference genome GRCm39 using HISAT2 (v2.2.1) [87]. Transcript assembly and quantification was carried out using StringTie (v2.2.1) [88], and DESeq2 was used for differential gene expression analysis applying a false discovery rate (FDR) threshold of 0.05. Gene set enrichment analysis was conducted using ShinyGO profiler (http://bioinformatics.sdstate.edu/go/) to detail GO enrichment pathways from KEGG, Reactome and WikiPathways, with 0.05 FDR cut off applied to denote significant pathways. All gene expression data is publicly available via Gene Expression Omnibus (GEO# GSE241404).

### Embryo retrieval and culture

Embryos (CD; *n* = 36 embryos from 5 litters, LPD; *n* = 45 embryos from 6 litters, MD-LPD; *n* = 42 embryos from 6 litters, WD; *n* = 51 embryos from 7 litters, MD-WD; *n* = 42 embryos from 6 litters, each generated by a separate male) were flushed from the oviduct at E1.5 using M2 media with 0.4% BSA (M7167—SigmaAldrich). Embryos are washed in pre-warmed EmbryoMax® KSOM media (MR-020P—Merk) and transferred to an EmbryoSlide (Virtolife) and cultured individually in EmbryoMax® KSOM media (37 °C; 5% CO_2_) overlaid with 1.4 ml EmbryoMax® mineral oil (ES-005—Merk). Embryos were cultured in an EmbryoScope time-lapse incubator for 60 h, with a 10-min image acquisition rate. Embryo cleavage rate and time taken to achieve developmental milestones was determined using EmbryoViewer software. Upon blastocyst expansion, image acquisition was stopped, and fully expanded blastocysts were fixed in 4% paraformaldehyde for staining of the inner cell mass and trophectoderm.

### Blastocyst inner cell mass and trophectoderm staining

Fixed cultured blastocysts were permeabilised, and unspecific binding was blocked using 0.1% Triton-X100, 3% normal goat serum and 1% BSA in PBS for 2 h at room temperature. Blastocysts were then incubated overnight at 4 °C in a primary antibody for Cdx2 (0.0775 μg/ml; ab157524; Abcam, UK) in PBS with 0.5% Triton-X100, 1% BSA. After blastocysts were washed in PBS containing 0.5% Triton-X100, 1% BSA they were incubated in 8 μgL/ml Alexa-Fluor conjugated secondary goat-anti-mouse antibody (ab150077; Abcam, UK), for 2 h at room temperature. Blastocysts were washed and re-blocked in 0.1% Triton-X100, 3% normal goat serum, 1% BSA, for 1 h, before incubation in a primary antibody for Oct4 (1.9 μg/ml; ab181557; Abcam, UK), made in PBS with 0.5% Triton-X100, 1% BSA, for 1.5 h at room temperature. Following washing, blastocysts were incubated with the appropriate Alexa Fluor conjugated secondary antibody (8 μgL/ml; ab175473; Abcam, UK) in PBS with 0.5% Triton-X100, 1% BSA, for 30 min at room temperature and counter stained with VECTASHIELD® Antifade Mounting Medium with DAPI (Vector Laboratories, UK). Blastocyst were imaged using a Nikon Eclipse 90i microscope using the Volocity imaging software to construct Z-stacked images captured in 2 μm intervals at × 40 magnification to image the whole blastocyst structure. Individual cells were counted as ICM if stained for Oct4 and TE if stained for Cdx2. CD *n* = 8 blastocysts from 5 litters, LPD *n* = 7 blastocysts from 6 litters, MD-LPD *n* = 7 blastocysts from 6 litters, WD *n* = 9 blastocysts from 7 litters and MD-WD *n* = 5 blastocysts from 6 litters, each litter generated by a separate male.

### Seminal vesicle fluid proteomics

Seminal vesicles were isolated from diet-fed males (aged 35–36 weeks, *n* = 6/diet group), and the contents were collected in 100 mM triethylammonium bicarbonate (TEAB) buffer with cOmplete™ ULTRA protease inhibitor (Roche, UK) and centrifuged to remove cellular debris; SDS was added to supernatant to achieve 5% final concentration. Seminal vesicle fluid was analysed by quantitative proteomic liquid chromatography mass spectrometry (LC-MSMS). Samples were digested via S-Trap (Protifi) using S-Trap micro spin columns and the standard manufacturer methodology ‘S-Trap Micro Long 4.7’ with the following modification to the start of the protocol—protein lysed samples (50 µg protein) were dried by spin concentrator/speedvac prior to the addition of 25 µL 5% SDS. The eluted peptides were dried and reconstituted in 30 µL mobile phase A (0.1% formic acid in water) and a calculated 6.67 ug (4 µL) injected via an Eksigent 425 LC system first through a trap column, 3 min at 10 µL/min 100% mobile phase A loading (YMC Triart 0.3 × 20 mm C18) before gradient elution (5 µL/min, column oven 35° C))onto the analytical column (150 × 0.3 mm YMC Triart C18) in line to a Sciex TripleTOF 6600 utilising the Duospray Source using a 50-μm electrode in positive mode, + 5500 V [89]. The following linear gradients were used: for SWATH, mobile phase B increasing from 3 to 30% over 38 min, 30% to 40% over 5 min, 40% to 80% over 2 min for wash and re-equilibration (total run time 57 min). SWATH acquisition used 100 variable windows optimised on sample type of 25 ms accumulation time following a single TOFMS scan for 50 ms (see table for SWATH window parameters) for a total cycle time of 2.6 s. SWATH data was assessed using DIA-NN v1.8 software [90] utilising an in silico library generated from the Proteome FASTA file (Mouse Swissprot canonical Accessed April 2022) to determine differentially expressed proteins. Data was processed, and only proteins detected in > 3 sample replicates were carried forward for subsequent analysis. Data was assessed for statistical significance using R-package Limma via the online StatsPro software [91], with pre-processing CV threshold of 0.5 and no threshold applied to fold change, Fisher test *padj* < 0.05 was deemed to be significant. The mass spectrometry proteomics data (acquisition files from the mass spectrometer and DIA-NN output files) have been deposited to the ProteomeXchange Consortium (http://proteomecentral.proteomexchange.org) via the PRIDE partner repository with the dataset identifier PXD044980, and the SWATH and DIANN parameters can also be found in Additional file 10. Data interpretations of differentially expressed proteins was conducted using free-web based software, Venny 2.1 [28], DAVID Bioinformatics Resources [92, 93] and UniProt ID mapping [94].

### Maternal uterine blood vessel staining

To identify blood vessels in the E3.5 uterus, formalin-fixed, paraffin embedded uterine horns (CD *n* = 7, LPD *n* = 5, MD-LPD *n* = 6, WD *n* = 6, MD-WD *n* = 6) were sectioned longitudinally and endothelial cells in blood vessels identified using a Blood Vessel Staining Kit (ECM590; EMD Millipore, Germany) as per the manufacturer’s protocol. The primary antibody used was anti-CD31 (ab28364, Abcam, UK) diluted 1:100 in PBS. Images were acquired using the Leica DMRB microscope Slide Scanner (Leica Biosystems, Germany), Retiga-2000R camera (QImaging, UK) and Surveyor computer software to image the whole section. The images were analysed by a blinded operator using ImageJ to determine the number of blood vessels in an area of between 1,000,000 and 1,500,000 μm^2^. Only vessels with a fully stained endothelial layer and visible lumen were used to calculate blood vessel perimeter and area.

### Uterine tissue gene expression

Uterine tissue total RNA was isolated from 10 to 15 mg E3.5 uterine tissue (CD *n* = 7, LPD *n* = 7, MD-LPD *n* = 7, WD *n* = 7, MD-WD *n* = 7) disrupted using TissueLyserII (2 cycles at 25 Hz for 30 s) using the RNeasy Mini kit (Qiagen, Germany) as per the manufacturer’s protocol for animal tissue and with additional on-column gDNA digestion using the RNase-free DNase set (Qiagen, Germany). RNA concentration was estimated using the NanoDrop (ND-1000) Spectrophotometer. cDNA synthesis was performed using the two-step precision NanoScript reverse transcription kit (PrimerDesign, UK), according to manufacturer’s protocol with 1 μg input of RNA. Real-time quantitative PCR was performed as previously outlined [95], to determine expression of genes stated in Additional file 11: Table S9. Briefly, triplicate reactions consisted of 5 ng cDNA and 175 nM forward and reverse primers (Eurofins Genomics, Germany) with 1 × Precision SYBR green Mastermix (PrimerDesign, UK), and RT-qPCR was performed using an Applied Biosystems 7500 system. Gene expression was analysed using the delta-delta Ct method relative to CD expression, with the GeNorm method used to normalise gene expression, as previously described [96] to two reference genes.

### Statistical analysis

All data were assessed for normality with GraphPad Prism (version 9) or SPSS (version 28). All stud male physiological data and maternal data were analysed using a one-way ANOVA for normally distributed data or a Kruskal–Wallis test for non-normally distributed data with appropriate post hoc test. *Z*-scores for male weight gain were calculated for each week using the equation [(*x*-*μ*)/*σ*]; where *x* = weight, *μ* = the geomean of all weights and *σ* = standard deviation of all weights. Male tissue weights were analysed using a one-way ANOVA with body weight at sacrifice included as a covariate. Embryo data were analysed using a multilevel random effects regression model with paternal origin of litter incorporated as the only random effect covariate using SPSS. For the analysis of serum lipid profiles, multiple ANOVA or Kruskal–Wallis tests were used to identify lipids, or lipid classes, of significantly different abundance between groups when individual variables were normally or non-normally distributed [97]. A Benjamini–Hochberg false discovery rate analysis was applied to the analysis of individual lipids, and significance was taken at *p* < 0.05. For the targeted analysis of tissue one-carbon metabolites, univariate analysis was performed in the R environment using the Univariate statistics function made available by Workflow4Metabolomics; *p* < 0.05 was used to identify features showing a significant difference in intensities between groups. False discovery rate (FDR) correction was performed to control the false discovery rate upon multiple testing, and a subsequent post hoc (Tukey, *p* < 0.05) test was applied to determine significant elevations/reductions in metabolites.

## Supplementary Information

Additional file 1: Figure S1. Principal component analysis plot of male serum lipids. PCA scores of the first two components for PCA models with QC samples demonstrating variability of male serum lipids detected using untargeted lipidomics in both positive and negative ion modes for all groups and quality control samples.

Additional file 2: Table S1. Full details of all stud male serum lipid changes. Lists of the differential (compared to CD) lipids and all detected lipids, including both positive and negative probabilistic quotient normalisation (PQN) values for each lipid and each biological replicate. (2_TableS1_Serum lipids.xls)

Additional file 3: Table S2. All significant differentially expressed genes found in stud male sperm RNASeq analysis. Details of all differentially expressed genes (DEGs) detected in male sperm compared to CD. (3_TableS2_SpermRNASeq_padj0.05.xls)

Additional file 4: Table S3. Predicted pathways influenced by the differentially expressed genes in male sperm. Details of all pathways predicted to be altered by changes in male sperm protein coding genes, detected using ShinyGO online software utilising KEGG Pathway, Reactome and WikiPathway repositories. (4_TableS3_SpermRNASEq_DEGs_All pathways.xls)

Additional file 5: Table S4. Effect of paternal diet on number of embryos, percentage blastocyst achievement and arrest (5_TableS4_Embryos.pdf)

Additional file 6: Table S5. All detected seminal vesicle fluid proteins. Full seminal vesicle fluid protein abundance details for all males and diet groups, including both unique and shared protein groups. (6_TableS5_SemVes Fluid Proteins.xls)

Additional file 7: Table S6. Detailed StatsPro output of seminal vesicle fluid protein comparisons. Individual male protein abundances from comparisons that had a differential protein expression between different diet groups and the StatsPro statistical outputs, including fill limma summary for all samples. (7_TableS6_StatsPro and limma output.xls)

Additional file 8: Table S7. Ingredients and nutritional information of diets fed to male mice. Full details of the custom diet formulations and additional components, all diets manufactured by Special Diet Services, WD available commercially with no customisation (diet code 829100). (8_TableS7_Diet composition.pdf)

Additional file 9: Table S8. Details of Multiple Reaction Monitoring (MRM) optimisation for targeted liver and testis metabolites. The optimised collision energy and RF lens voltage for the optimised MRM transitions for each analyte (9_One Carbon Metabolism.docx)

Additional file 10. SWATH and DIANN Parameters. Specific details relating to the ultra-performance liquid chromatography-mass spectrometry (UPLC-MS) analysis parameters for proteins from male seminal vesicle fluid. (10_Proteomics.xls)

Additional file 11: Table S9. Details of primers used for RT-qPCR. Full details of experimental and control primer sequences used for detection of uterine gene expression via SYBR based RT-qPCR. All purchased from Eurofins Genomics. (12_TableS8_Primers.pdf)

## Data Availability

All data generated or analysed during this study are included in this published article and its additional information files and are available on figshare [98]. All RNA-Seq data has been deposited in NCBI’s Gene Expression Omnibus under accession number: GSE241404 (https://www.ncbi.nlm.nih.gov/geo/query/acc.cgi?acc=GSE241404) [99]. All proteomics mass-spectrometry data and processed results have been deposited on PRIDE (EMBL-EBI) repository under the accession number: PXD044980 (https://www.ebi.ac.uk/pride/archive/projects/PXD044980) [100]. The metabolite datasets of male liver and testis can be found on figshare (10.6084/m9.figshare.26326309.v2) [98]. The entire serum lipidomic dataset is included as Additional file 2.

## Abbreviations

CD: Control diet
DEG: Differentially expressed gene
DEP: Differentially expressed protein
E: Embryonic day
FDR: False discovery rate
FC: Fold change
ICM: Inner cell mass
ISTD: Internal standard
LC-MSMS: Liquid chromatography mass spectrometry
LPD: Low-protein diet
MD-LPD: Methyl donor supplemented low-protein diet
MD-WD: Methyl donor supplemented Western diet
MRM: Multiple reaction monitoring
padj: P-adjusted
PCA: Principal components analysis
PQN: Probabilistic quotient normalisation
QA/QC: Quality assurance and quality control
QC: Quality control
SAM: S-(5′-adenosyl)-L-methionine
SAH: S-adenosylhomocysteine
TEAB: Triethylammonium bicarbonate
TE: Trophectoderm
UPLC-MS: Ultra-performance liquid chromatography-mass spectrometry
WD: Western diet
