# Supplementary material for: Paternal undernutrition and overnutrition modify semen composition and preimplantation embryo developmental kinetics in mice
Source: BMC Biol. 2024 Sep 16;22:207. doi: 10.1186/s12915-024-01992-0 (PMC11403970; doi:10.1186/s12915-024-01992-0)
Supplement: Supplementary file 1 — Additional file 1: Figure S1. Principal component analysis plot of male serum lipids. PCA scores of the first two components for PCA models with QC samples demonstrating variability of male serum lipids detected using untargeted lipidomics in both positive and negative ion modes for all groups and quality control samples. [file 12915_2024_1992_MOESM1_ESM.pdf]

Supplementary Figure 1

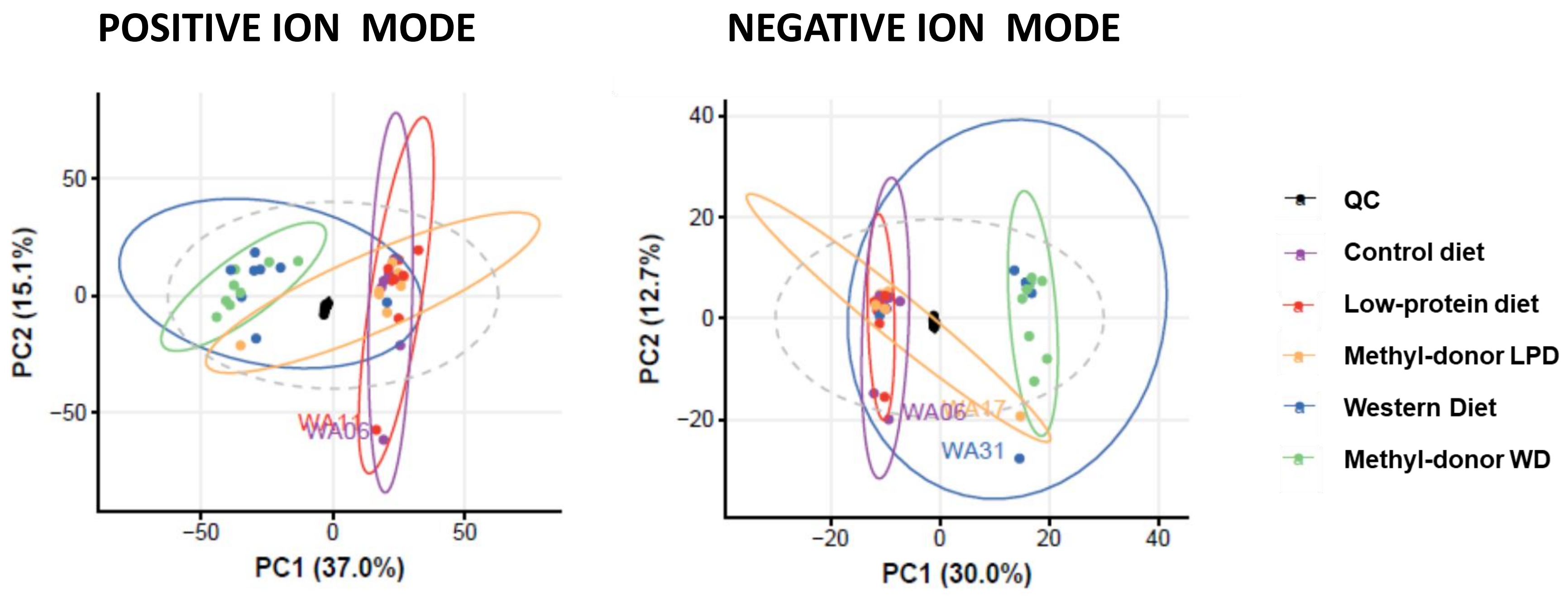

PCA scores of the first two components for PCA models with QC samples demonstrating variability of male serum lipids detected using untargeted lipidomics.
