# Supplementary material for: Paternal undernutrition and overnutrition modify semen composition and preimplantation embryo developmental kinetics in mice
Source: BMC Biol. 2024 Sep 16;22:207. doi: 10.1186/s12915-024-01992-0 (PMC11403970; doi:10.1186/s12915-024-01992-0)
Supplement: Supplementary file 5 — Additional file 5: Table S4. Effect of paternal diet on number of embryos, percentage blastocyst achievement and arrest (5_TableS4_Embryos.pdf) [file 12915_2024_1992_MOESM5_ESM.pdf]

Table 1: Effect of paternal diet on number of embryos, percentage blastocyst achievement and arrest.

		CD	LPD	MD-LPD	WD	MD-WD
Number of embryos flushed per litter		8.20 ±1.0	8.20 ±0.2	7.67 ±0.7	8.14 ±0.3	7.50 ±0.9
% Blastocyst development		82.9 ±10.5	91.0 ±3.9	93.8 ±6.3	90.1 ±6.7	90.5 ±9.5
% Full expansion		91.1 ±6.5	92.8 ±4.9	87.8±5.8	98.4 ±1.6	89.0 ±5.6
% Arrested	Pre-blastocoel	17.1 ±10.5	9.0 ±3.9	6.2 ±6.3	9.9 ±6.7	9.5 ±9.5
	Post-blastocoel	8.9 ±6.5	7.2 ±4.9	12.2 ±5.8	1.6 ±1.6	11.0 ±5.6

Data presented as litter average (±SEM) for number of embryos flushed, and as average of all embryos assessed (±SEM) for percentages. n=44-53 embryos (from 6-7 litters, each generated by a separate male).
