# Supplementary material for: Paternal undernutrition and overnutrition modify semen composition and preimplantation embryo developmental kinetics in mice
Source: BMC Biol. 2024 Sep 16;22:207. doi: 10.1186/s12915-024-01992-0 (PMC11403970; doi:10.1186/s12915-024-01992-0)
Supplement: Supplementary file 8 — Additional file 8: Table S7. Ingredients and nutritional information of diets fed to male mice. Full details of the custom diet formulations and additional components, all diets manufactured by Special Diet Services, WD available commercially with no customisation (diet code 829100). (8_TableS7_Diet composition.pdf) [file 12915_2024_1992_MOESM8_ESM.pdf]

Supplemental Table 7: Ingredients and nutritional information of diets fed to male mice

	CD	LPD	MD-LPD	WD†	MD-WD
**Energy density (kcal/g)**	3.10	3.10	3.10	4.63	4.63
**Proportional energy content (%)**					
Protein	16	8.4	8.4	14.7	14.7
Fat	23.4	23.1	23.1	41.4	41.4
Carbohydrate	60.5	68.5	68.5	43.9	43.9
of which sugars	*20.7*	*23.6*	*23.6*	*34.6*	*34.6*
**Protein (% g/g)**					
Casein	18.0	9.0	9.0	19.5	19.5
**Fats (% g/g)**					
Corn Oil	10	10	10	1	1
Milk fat	-	-	-	20	20
Cholesterol	-	-	-	0.15	0.15
**Carbohydrates (% g/g)**					
Sucrose	21.3	24.3	24.3	33.9	33.9
Starch Maize	42.5	48.5	46.0	15.0	12.3
Cellulose	5	5	5	5	5
**Micronutrients (% g/g)**					
Choline chloride	0.2	0.2	0.7	0.2	0.7
D,L-Methionine	0.50	0.50	1.25	0.00	0.75
Betaine	-	-	1.5	-	1.5
Mineral mix (AIN-76)	2	2	2	3.5	3.5
Vitamin mix (AIN-76)	0.5	0.5	0.5	1.0	1.0
*Folic Acid	$1x10^{-4}$	$1x10^{-4}$	$1.6x10^{-3}$	$2x10^{-4}$	$1.6x10^{-6}$
*Vitamin B12	$5x10^{-7}$	$5x10^{-7}$	$1.5x10^{-4}$	$1x10^{-6}$	$1.5x10^{-4}$

* Components contained within commercially available Vitamin mix AIN-76 that were supplemented in methyl-donor diets.

† WD is commercially manufactured by Special Diet Services, diet code 829100. All other diets are custom formulations from Special Diet Services.
