# Supplementary material for: Paternal undernutrition and overnutrition modify semen composition and preimplantation embryo developmental kinetics in mice
Source: BMC Biol. 2024 Sep 16;22:207. doi: 10.1186/s12915-024-01992-0 (PMC11403970; doi:10.1186/s12915-024-01992-0)
Supplement: Supplementary file 9 — Additional file 9: Table S8. Details of Multiple Reaction Monitoring (MRM) optimisation for targeted liver and testis metabolites. The optimised collision energy and RF lens voltage for the optimised MRM transitions for each analyte (9_One Carbon Metabolism.docx) [file 12915_2024_1992_MOESM9_ESM.docx]

*MRM Optimisation*

Multiple Reaction Monitoring (MRM) optimisation was performed using the automated function in TSQ Quantiva Tune 2.0 by direct infusion of individual standards (25-50µg.mL^-1^). The optimised collision energy and RF lens voltage for the optimised MRM transitions for each analyte are shown in the table below.

| Compound | Ion | Precursor (m/z) | Product (m/z) | Collision Energy (V) | RF Lens (V) |
| --- | --- | --- | --- | --- | --- |
| DL Homocysteine | Quan | 136.039 | 46.986 | 28.809 | 42.854 |
| DL Homocysteine | Qual | 136.039 | 55.986 | 17.736 | 42.854 |
| Homocysteine-d_4_ | Quan | 140.039 | 48.929 | 29.517 | 41.618 |
| Homocysteine-d_4_ | Qual | 140.039 | 58.986 | 18.949 | 41.618 |
| Methionine | Qual | 150.07 | 104.000 | 10.253 | 44.337 |
| Methionine | Quan | 150.07 | 133.000 | 10.253 | 44.337 |
| Methionine-(methyl ^13^C_3_) d_3_ | Qual | 154.283 | 107.986 | 10.253 | 43.843 |
| Methionine-(methyl ^13^C_3_) d_3_ | Quan | 154.283 | 137.000 | 10.253 | 43.843 |
| Pyridoxine | Quan | 170.117 | 134.040 | 20.618 | 58.180 |
| Pyridoxine | Qual | 170.117 | 152.040 | 12.427 | 58.180 |
| Pyridoxine-d_2_ | Quan | 172.07 | 136.058 | 21.933 | 55.213 |
| Pyridoxine-d_2_ | Qual | 172.07 | 154.058 | 13.691 | 55.213 |
| SAH | Qual | 385.191 | 88.000 | 37.556 | 74.000 |
| SAH | Quan | 385.191 | 136.040 | 20.011 | 74.000 |
| SAH-d_4_ | Qual | 389.217 | 92.000 | 39.528 | 71.034 |
| SAH-d_4_ | Quan | 389.217 | 138.071 | 19.961 | 71.034 |
| SAM | Qual | 399.23 | 136.04 | 26.18 | 75.483 |
| SAM | Quan | 399.23 | 250.111 | 14.904 | 75.483 |
| Folic Acid | Qual | 442.222 | 176.040 | 37.910 | 67.000 |
| Folic Acid | Quan | 442.222 | 295.040 | 15.966 | 67.000 |
| folic acid-(glutamic acid-^13^C_5_,^15^N) | Qual | 448.183 | 176.375 | 39.124 | 65.348 |
| folic acid-(glutamic acid-^13^C_5_,^15^N) | Quan | 448.183 | 295.129 | 18.697 | 65.348 |
| 5MTHF | Qual | 460.27 | 194.111 | 32.551 | 77.708 |
| 5MTHF | Quan | 460.27 | 313.111 | 19.303 | 77.708 |
| Vitamin B12 | Quan | 678.461 | 147.111 | 39.022 | 112.562 |
| Vitamin B12 | Qual | 678.461 | 359.111 | 22.539 | 112.562 |
