# Supplementary material for: Paternal undernutrition and overnutrition modify semen composition and preimplantation embryo developmental kinetics in mice
Source: BMC Biol. 2024 Sep 16;22:207. doi: 10.1186/s12915-024-01992-0 (PMC11403970; doi:10.1186/s12915-024-01992-0)
Supplement: Supplementary file 11 — Additional file 11: Table S9. Details of primers used for RT-qPCR. Full details of experimental and control primer sequences used for detection of uterine gene expression via SYBR based RT-qPCR. All purchased from Eurofins Genomics. (12_TableS8_Primers.pdf) [file 12915_2024_1992_MOESM11_ESM.pdf]

Supplemental Table 7: RT-qPCR primer details

Gene	Full name	Accession code	Primer Sequences (5'-3')		Amplicon Length (nt)
			Forward Primer	Reverse Primer	
**Cd14**	CD14 antigen	NM_009841.4	aaagaaactgaagcctttctcg	agcaacaagccaagcacac	89
**Itgam**	Integrin alpha M	NM_001082960.1	agccccacactagcatcaa	tccatgtccacagagcaaag	73
**Itgax**	Integrin alpha X	NM_021334.3	agcctcaagacaggacatcg	tgaatcctggaggggatct	72
**Ptges**	Prostaglandin E synthase	NM_022415.3	gcacactgctggtcatcaag	acgtttcagcgcatcctc	101
**Ptgs1**	Prostaglandin-endoperoxide synthase 1	NM_008969.3	cctctttccaggagctcaca	tcgatgtcaccgtacagctc	70
**Ptgs2**	Prostaglandin-endoperoxide synthase 2	NM_011198.3	gggagtctggaacattgtgaa	gtgcacattgtaagtaggtggac	112
**Vegfa**	Vascular endothelial growth factor a	NM_001025250.3	actggaccctggctttactg	tctgctctccttctgtcgtg	78
**Ppiβ**	Peptidyl-Prolyl Cis-Trans Isomerase β	NM_011149	ttcttcataaccacagtcaagacc	accttccgtaccacatccat	92
**Tbp**	TATA-Box Binding Protein	NM_013684.3	gggagaatcatggaccagaa	gatgggaattccaggagtca	90
